# Synthesis of thermostable artificial nicotinamide cofactors: carba-NAD^+^ and carba-NADP^+^

**DOI:** 10.1039/d6cb00036c

**Published:** 2026-05-05

**Authors:** Zinnia Dsouza, Jan-Simon Jeshua Friedrichs, Manuel Döring, Nicolás Rincón Téllez, Luca Schmermund, Volker Sieber

**Affiliations:** a Chair of Chemistry of Biogenic Resources, Technical University of Munich, Campus Straubing, Schulgasse, 16 94315 Straubing Germany sieber@tum.de; b Catalytic Research Center, Technical University of Munich, Ernst-Otto-Fischer-Straße 1 85748 Garching Germany; c SynBiofoundry@TUM, Technical University of Munich, Schulgasse 22 94315 Straubing Germany; d School of Chemistry and Molecular Biosciences, The University of Queensland, 68 Copper Road St. Lucia 4072 Australia

## Abstract

Carbocyclic nicotinamide cofactors are attractive NAD(P)^+^ analogues that retain native redox activity while offering substantially enhanced chemical and thermal stability. Their broader use, however, has been limited by demanding multistep chemical syntheses involving complex protection strategies and limited exploration of enzymatic alternatives. In this study, we studied both chemical and enzymatic approaches for the synthesis of carba-analogues. For the enzymatic route, we identify and characterize the key enzymes involved in cofactor assembly, evaluating their tolerance toward non-native, carbocyclic substrates and extending the assembly to further generate the phosphorylated analogue, cNADP^+^. In addition, extending the analysis to cofactor thermostability, carba-NAD^+^ displayed a remarkable halftime (*t*_1/2_ > 1386 h at 50 °C), far exceeding that of NAD^+^ (*t*_1/2_ = 76 h) and is accepted by a broad panel of oxidoreductases. Collectively, this work outlines a modular workflow and details the synthesis landscape for accessing thermostable, synthetic nicotinamide cofactor analogues, cNAD^+^ and cNADP^+^, unveiling new opportunities for their application in cofactor engineering and synthetic biocatalysis.

## Abstract 2

Carbocyclic nicotinamide cofactors are synthesized *via* chemical and enzymatic routes, with strategies compared to improve access to costly biomimetics. These artificial cofactors exhibit high thermostability and broad oxidoreductase compatibility.

## Graphical abstract

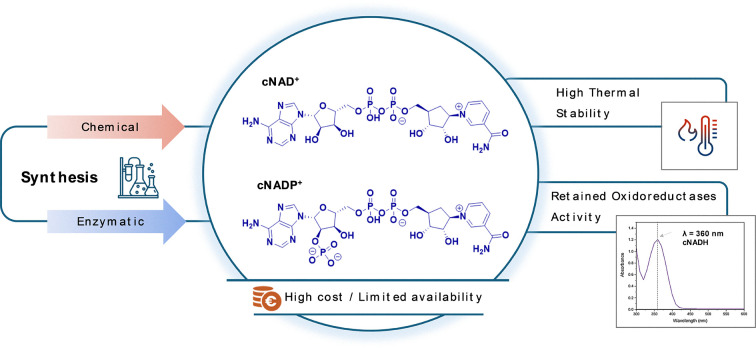

## Introduction

Nicotinamide cofactors such as nicotinamide adenine dinucleotide (NAD^+^) and its phosphate derivative (NADP^+^) are indispensable in redox biocatalysis, enabling oxidoreductases to mediate a multitude of redox transformations integral to metabolism and industrial biocatalysis.^1,2^ However, their high cost and chemical instability limit their use in industrial biocatalysis, confining many processes to moderate temperatures. More than 15 000 sequences have been annotated as NAD(P)^+^- or NAD(P)H-utilizing enzymes;^3^ yet, the inherent fragility of these cofactors continues to constrain the industrial application of oxidoreductases, especially when compared with the broader adoption of cofactor-independent hydrolases and oxidases.^4,5^

Although numerous strategies have been developed and assessed to mitigate the cost-related challenges in industrial biocatalysis through cofactor recycling,^6^ the thermal sensitivity of NAD(P)H, particularly its tendency to degrade under elevated temperature process conditions, remains a significant bottleneck and prohibits extending redox catalysis to higher temperatures.^7^ Previous studies have examined the thermal degradation of NAD^+^*in vitro*.^8^ Under strongly elevated temperatures (100 °C) in 0.1 M Tris buffer at pH 7.6, NAD^+^ displays a half-life of roughly 10 minutes, corresponding to a degradation rate constant (*k*_d_) of 0.050 min^−1^. The decomposition proceeds primarily through cleavage of the nicotinamide–ribose linkage, generating nicotinamide and ADP-ribose as the principal products.^9^

Meanwhile, rapid advances in enzyme engineering have begun to tailor biocatalysts to accept non-canonical cofactors, fuelling parallel progress in cofactor engineering. This synergy has accelerated the development and exploration of synthetic cofactor mimetics as powerful tools for elucidating metabolic pathways and enabling the design of next-generation biocatalytic systems.^10–12^ Alongside these advances, minimizing production costs remains a central objective in scaling up enzymatic processes. To address these combined challenges, synthetic nicotinamide cofactors have been developed and studied to enhance stability, expand catalytic flexibility, and enable non-natural enzymatic transformations, ultimately expanding the potential of biocatalysis beyond traditional biochemical pathways.^12–16^

In an early work, carba-NAD(P)^+^ (cNAD(P)^+^), a synthetic analog of NAD(P)^+^ was introduced as an NAD(P)^+^ mimetic in which the β-d-ribose ring is replaced with a 2,3-dihydroxycyclopentane moiety.^17^ This structural modification was found to enhance thermostability, making the cofactor resistant to hydrolysis at elevated temperatures, as specifically shown for NADP^+^.^13^ The increased stability reduces the need for frequent replenishment, lowering operational costs and supporting continuous, scalable bioprocesses, making the analog particularly attractive for industrial applications. Originally designed by Slama and Simmons as a specific inhibitor to probe the biological roles of NAD-glycohydrolases and ADP-ribosyltransferases,^17^ carba-NAD(P)^+^ later emerged as a promising substitute for NAD(P)/H in glucose sensing, demonstrating superior performance and application potential.^18^ A patented method by Roche Diagnostics further underscored the practical value of cNAD^+^, showing significantly improved enzyme stability when stored with this robust coenzyme.^19^ This advancement enabled enzymatic reduction reactions utilizing carba-cofactors, exemplified by glucose dehydrogenase. Additionally, enzyme engineering was used for the first time to generate enzyme variants with improved acceptance of carba-cofactors.^20^ Recognizing its biological significance, an improved chemical synthesis of cNAD^+^ was later developed employing six steps, employing a pyrophosphate coupling strategy that achieved a yield of 58%.^21^ Synthesized cNAD^+^ and related NAD^+^ mimetics have also been demonstrated to explore their inhibitory activity against ADP-ribosyltransferase activity.^22^

While the biochemical relevance of cNAD(P)^+^ is increasingly recognized, its broader application remains limited by the complexity of chemical synthesis and the underexplored development of enzymatic production methods. Chemical routes typically begin with commercially available precursors, allowing for precise structural and functional modifications. However, these processes are intricate, labor-intensive, requiring tightly controlled reaction conditions and specialized reagents, and can suffer from reduced overall yields due to side reactions and intermediate instability.^23^ As a result, large-scale preparation is costly and of limited practicality. Currently, carbocyclic nicotinamide cofactors are offered only by a few specialty suppliers at high cost (*e.g.*, cNAD^+^ ≈ €200 per mg vs NAD^+^ ≈ €100 per g, Sigma-Aldrich, accessed April 10, 2026), and the phosphorylated analogue carba-NADP^+^ is scarcely available. These constraints restrict their routine use in enzymatic and biocatalytic studies and highlight the need for efficient and accessible alternatives. Developing and studying complementary enzymatic approaches therefore hold both practical and methodological significance.

In general, enzymatic synthesis is preferred over chemical methods for its environmental and cost advantages, operating under milder conditions (*e.g.*, optimized pH and temperature), requiring fewer reagents and toxic chemicals, and providing high selectivity and precise control over regio- and stereochemistry, which enhances purity and process efficiency.^24,25^ The alternative synthesis of cNAD^+^, involving two late-stage sequential enzymatic steps and their respective enzyme classes, has been outlined in a published patent.^26^ A closer look at NAD^+^ biosynthesis reveals two converging metabolic routes: the *de novo* and salvage pathways. In the salvage pathway, nicotinamide riboside (NR), a form of vitamin B3, is first phosphorylated by nicotinamide riboside kinase (NRK) to form nicotinamide mononucleotide (NMN), which is then converted to NAD^+^ by NMN adenylyltransferase (NMN-AT) through the transfer of an adenylyl group from ATP.^27,28^ Notably, both NRK and NMN-AT have been claimed to accept carba-analogues as substrates.^26^

While the enzymes involved in NAD^+^ metabolism are well-characterized,^29,30^ their ability to process carba-analogues has not been systematically investigated. This gap underscores the need for defined enzyme variants and mechanistically characterized reactions capable of supporting enzymatic cNAD^+^ synthesis. Building on established knowledge of NAD^+^ biosynthetic pathways, we aimed to examine whether key enzymes involved in cofactor metabolism can accept carbocyclic nicotinamide substrates, to characterize their catalytic behaviour, and to explore the feasibility of a biocatalytic route to cNAD^+^. In this context, we also investigated the capacity of NAD^+^ kinases to act on cNAD^+^ and showed that phosphorylation at the 2′-position could be achieved enzymatically to form cNADP^+^, thereby extending enzymatic access to carbocyclic nicotinamide cofactors. While the enzymatic conversion of NAD^+^ to NADP^+^ is well-documented,^31^ chemical methods are less common.

Comprehensive enzyme characterization is a prerequisite for the development of selective and reproducible biocatalytic transformations. In this work, we report the biochemical characterization of key enzymes and evaluate their catalytic performance under preparative conditions to enable enzymatic access to cNAD^+^ and cNADP^+^. The following sections describe the chemical and enzymatic synthesis routes, the thermal stability of cNAD^+^, and its functional evaluation with oxidoreductases, thereby extending our previous work on carbocyclic cofactors^13,20^ and providing a foundation for their broader application in biocatalysis and cofactor engineering.

## Results and discussion

As a preliminary step, we reproduced the literature-established chemical synthesis of carba-NAD^+^ to later serve as a reference standard for enzymatic pathway evaluation. The previously developed multi-step synthetic route was implemented following established protocols with minor procedural optimizations for yield and purity.

### Chemical synthesis of cNAD^+^

**Fig. 1:**
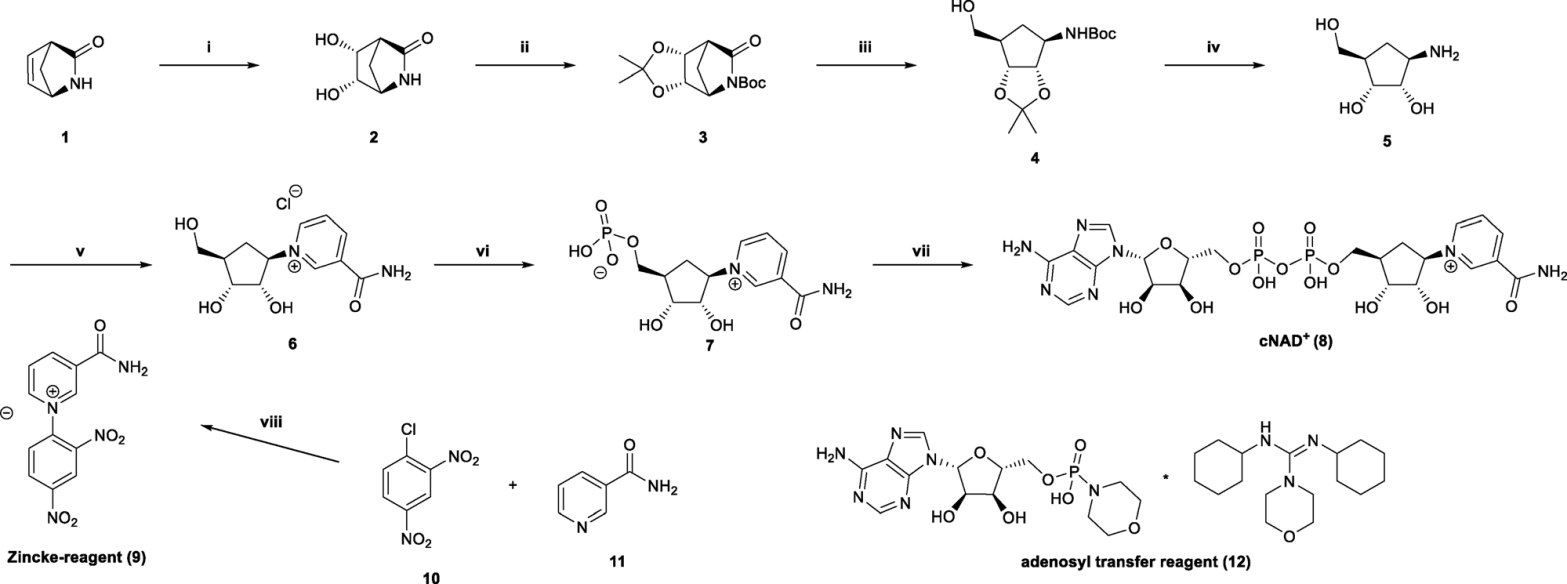
Chemical synthesis of carba-nicotinamide adenine dinucleotide (cNAD^+^). The pathway proceeds from the aminocyclopentane precursor (1*R*,2*S*,3*R*,5*R*)-3-amino-5-(hydroxymethyl)cyclopentane-1,2-diol. Coupling with the pyridinium compound (Zincke reagent) affords carba-nicotinamide riboside (cNR), which is phosphorylated at the 5′-position to yield carba-nicotinamide mononucleotide (cNMN). Subsequent coupling with adenosine transfer reagent (adenosine 5′-monophosphomorpholidate 4-morpholine-*N*,*N*′-dicyclohexylcarboxamidine salt) leads to the formation of cNAD^+^. Yields for each step are indicated below.

In the past, several synthetic routes to obtain cNAD^+^ have been published.^17,21,32^ The main starting point of each published stereospecific synthetic route, as well as of the route presented here, is the commercially available (−)-2-azabicyclo [2.2.1] hept-5-en-3-one 1, also referred to as *R*-(−)-Vince lactam^21^ (Fig. 1).

The eight-step synthesis pathway presented here begins with the *syn*-dihydroxylation of Vince lactam using catalytic amounts of OsO_4_ and *N*-methylmorpholine *N*-oxide as the oxidant in stoichiometric amounts, providing the dihydroxy lactam 2 in 73% yield.^33^ Following the protocol described by Blackburn *et al.*, the vicinal diol moiety of dihydroxy lactam 2 is protected as a ketal using 2,2-dimethoxypropane, followed by the introduction of a *tert*-butyloxycarbonyl (BOC) protecting group into the lactam nitrogen. The protected lactam 3 was obtained in 90% yield over two steps.^32^

Lactam 3 then undergoes facile reductive ring-opening using excess sodium borohydride in MeOH, giving the BOC-protected amine 4 in 84% yield. Subsequently, the BOC and ketal protecting groups are cleaved using aqueous hydrogen chloride in a 1 : 1 mixture of THF and MeOH,^34^ affording the deprotected amine 5 in 98% yield. The reductive ring-opening involving the protected lactam 3, rather than the unprotected lactam 2, has a significant advantage in that the use of toxic hydrogen fluoride to cleave borane complexes, as described by Szczepankiewicz *et al.*, can be avoided.^21^

With amine 5 in hand, one of the key steps in the synthetic route, namely the Zincke reaction, was performed to obtain carbanicotinamide riboside (cNR) 6 in 76% yield.^21^ To obtain pure cNR without any traces of solvents such as HOAc and MeOH carried over from column chromatography, the product was further purified by anion-exchange chromatography using a DOWEX 1×8 resin (chloride form).

Phosphorylation of cNR to carbanicotinamide mononucleotide (cNMN) 7 was performed using phosphoryl trichloride in trimethyl phosphate. Purification by cation-exchange chromatography (DOWEX 50W-X8, protonated form) provided cNMN as the inner salt in 88% yield.

The final step involved a diphosphate coupling reaction using the adenosyl transfer reagent adenosine 5′-monophosphomorpholidate 4-morpholine-*N*,*N*′-dicyclohexylcarboxamidine salt 12, together with manganese(ii) chloride and cNMN in formamide, to obtain cNAD^+^ with an isolated yield of 61%. High-purity cNAD^+^ was obtained *via* ion-exchange chromatography utilizing a DOWEX 1×8 resin in its formate form. The use of DOWEX 1×8 (formate form, generated from the chloride form using 2.5 M NaOH and 3 M formic acid) improved yield and simplified purification. Using this resin, the product was eluted under mild conditions with a 0.03 M solution of formic acid.

Chemical synthesis of CarbaNAD^+^. (i) OsO4, NMO, *tert*-BuOH, H_2_O, 70 °C, 4 h, 73%; (ii) (1) 2,2-dimethoxypropane, *p*-toluene sulfonic acid, DCM, rt, 24 h; (2) di-*tert*-butyldicarbonate, 4-(dimethylamino)pyridine, DCM, rt, 4 h, 91% over 2 steps; (iii) NaBH4, THF, 0 °C – rt, 24 h, 84%; (iv) 6 M HCl, MeOH/THF (1 : 1), rt, 24 h, 98%; (v) Zincke-reagent (9), NaOAc, MeOH, rt, 2 h, 76%; (vi) POCl3, PO(OMe)3, 0 °C, 88%; (vii) adenosine 5′-monophosphomorpholidate 4-morpholine-*N*,*N*′-dicyclohexylcarboxamidine salt (12), 0.5 M MnCl_2_ in formamide, rt, 24 h, 39%; (viii) 100 °C, 20 min, 61%.

**Fig. 2:**
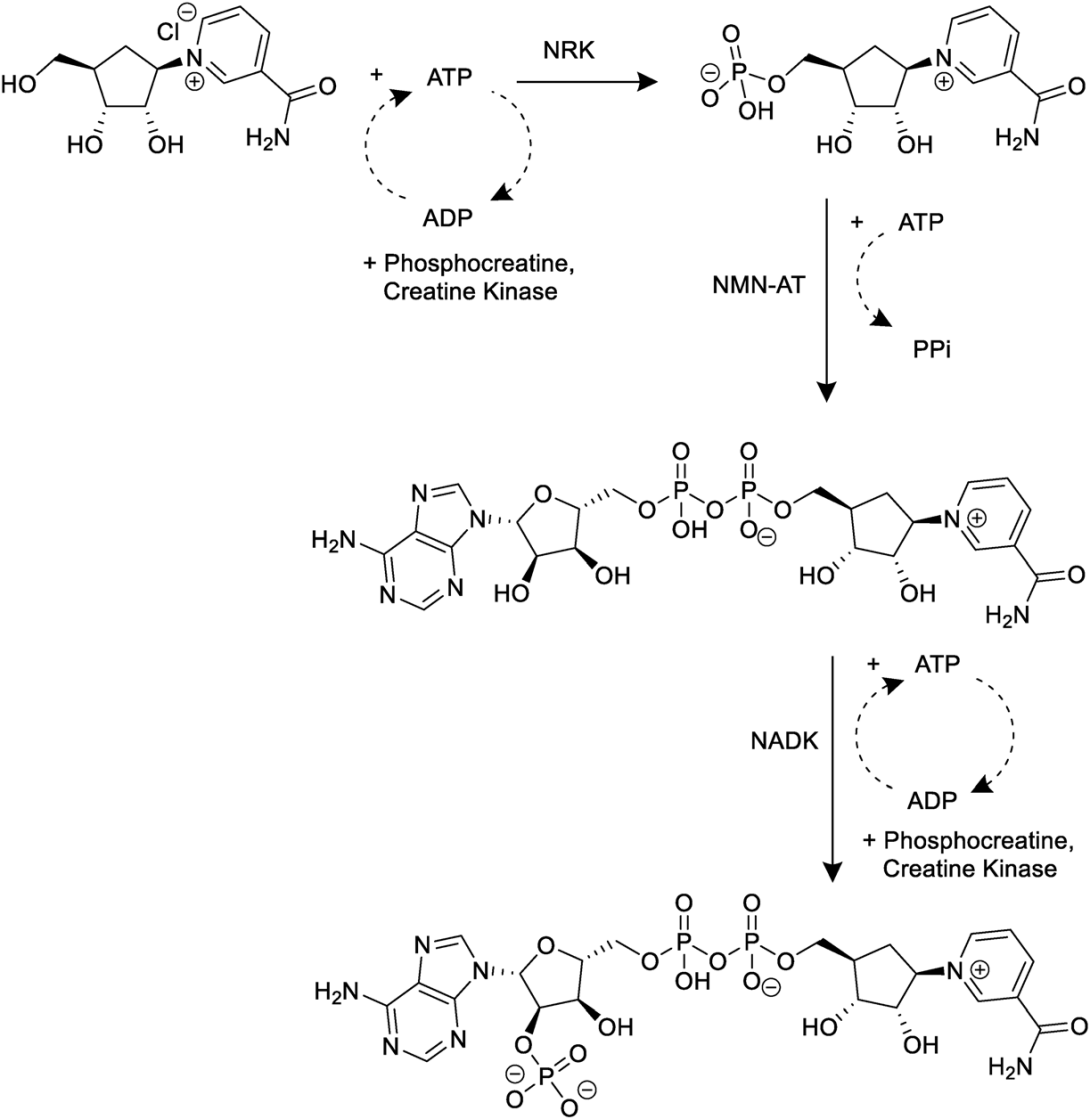
Enzymatic conversion of carba-nicotinamide riboside (cNR) to carba-nicotinamide adenine dinucleotide phosphate (cNADP^+^). NRK catalyzes ATP-dependent phosphorylation to yield cNMN; NMN-AT mediates adenylyl transfer to form cNAD^+^; NADK phosphorylates cNAD^+^ to cNADP^+^.

Although chemical synthesis provides reliable access to carbocyclic intermediates, its multistep nature and dependence on protecting-group chemistry motivate enzymatic alternatives for late-stage functionalization. In particular, the phosphorylation and adenylylation steps convert carba-nicotinamide riboside (cNR) to carba-NAD^+^ and carba-NADP^+^. We therefore examined enzymes from the corresponding biosynthetic classes for their ability to mediate the sequential conversion of cNR to carba-nicotinamide mononucleotide (cNMN), cNMN to cNAD^+^, and cNAD^+^ to cNADP^+^ (Fig. 2).

### Identification and characterization of enzymes

The enzymes were cloned, expressed, and purified, and their activity profiles were mapped across pH and temperature to define operational ranges. Steady-state kinetic parameters with the carba-substrates were then determined and compared to those of the native counterparts to quantify substrate acceptance and evaluate relative catalytic competence. Chemically synthesized intermediates (cNR, cNMN, and cNAD^+^) served as substrates for these assays.

#### Enzyme selection

To identify suitable enzymes for each of the three key biotransformations, two candidate enzymes were selected per the reaction step from the relevant enzyme classes (Table 1). The selection strategy combined an *Escherichia coli* homolog, chosen for its expected soluble expression, with a phylogenetically distinct variant to explore sequence–function diversity.

**Table 1:** Enzymes selected for the enzymatic conversion of cNR to cNADP^+^. Enzymes investigated in this study, including their source organisms, putative catalytic functions with corresponding enzyme commission (EC) numbers, and UniProt accession identifiers

	Enzyme	Source organism	Putative Function, EC No.	Uniprot ID.
(a)	*Ec*NRK	*Escherichia coli*	Nicotinamide riboside kinase 2.7.1.22	P27278
	*Ss*NRK	*Streptococcus sanguinis*		A3CQV5
(b)	*Ec*NMN-AT	*Escherichia coli*	Nicotinamide mononucleotide adenylyl transferase 2.7.7.1	P0A752
	*Ck*NMN-AT	*Citrobacter koseri*		A8AJG3
(c)	*Ec*NADK	*Escherichia coli*	NAD^+^ kinase 2.7.1.23	P0A7B3
	*PyH*NADK	*Pyrococcus horikoshii*		O58801

The alternative homologs were identified using BLAST searches in UniProt using *E. coli* reference sequences as queries. For nicotinamide riboside kinases (NRKs), an enzyme from *Streptococcus sanguinis* (*Ss*NRK) sharing 25% sequence identity with the *E. coli* homolog (*Ec*NRK) was selected to assess whether increased sequence divergence influences substrate acceptance or catalytic behaviour toward the carbocyclic analogue. This variant has not been previously characterized with the native substrate, requiring an initial biochemical evaluation. For nicotinamide mononucleotide adenylyltransferases (NMN-ATs), a putative enzyme from *Citrobacter koseri* (*Ck*NMN-AT) with 84.5% sequence similarity to *E. coli* NMN-AT (*Ec*NMN-AT) was chosen due to the absence of prior functional data. Enzymes *Ss*NRK and *Ec*NMN-AT have previously been reported in the patent literature as suitable enzymes for carbocyclic cofactor synthesis.^26^ For the NAD^+^ kinase-mediated phosphorylation step, a homolog from the thermophilic archaeon thermophilic archaeon *Pyrococcus horikoshii* (*PyH*NADK) was chosen. Rather than limiting the screen to closely related homologs, sequence diversity was deliberately explored to balance functional predictability with the potential to reveal differences in substrate tolerance across enzyme families.

#### Expression and biochemical characterization of enzymes

All selected enzyme candidates were obtained in the soluble form and exhibited the expected molecular weights based on SDS–PAGE analysis (Fig. S1). Size-exclusion chromatography revealed distinct quaternary organization among the homologs: NRK variants predominantly assembled tetramers, NMNATs were monomeric, and NAD kinases displayed as a hexamer and tetrameric assemblies (Fig. S2).

#### Steady-state kinetic characterization

Steady-state kinetic analyses were performed to assess the substrate acceptance and catalytic behaviour of the selected enzymes toward both native and carbocyclic analogues (Table 2). Kinetic parameters were determined using coupled spectrophotometric assays, in which product formation was linked to NAD(P)H turnover and was monitored *via* changes in absorbance. For NRKs, both *Ec*NRK and *Ss*NRK showed comparable *K*_M_ values for the natural NR (∼1–1.7 mM) and the carbocyclic analog cNR, with modest increased *K*_M_ values observed for *Ss*NRK. Catalytic turnover (*k*_cat_) was higher for *Ec*NRK across both substrate types, reaching 3.34 s^−1^ for NR and 1.17 s^−1^ for cNR. The modest drop in *k*_cat_ with cNR indicates that substitution of the ribose ring with a carba moiety does not significantly impair catalytic function in NRKs.

**Table 2:** Steady-state kinetic parameters of NRK, NMN-AT, and NADK enzymes with native and carbocyclic substrates. Values represent mean ± standard deviation from triplicate determinations. Kinetic parameters were determined under saturated ATP concentrations and fitted to the Michaelis–Menten equation

Enzyme	*K*_M_ substrate (mM)	*K*_M_ cofactor (mM) ATP	*V*_max_ (U mg^−1^)	*k*_cat_ (s^−1^)
NR to NMN				
*Ec*NRK	1.02 ± 0.04	1.37 ± 0.04	4.23 ± 0.07	3.34
*Ss*NRK	1.71 ± 0.16	2.55 ± 0.26	2.21 ± 0.07	1.58

cNR to cNMN				
*Ec*NRK	1.0 ± 0.07	1.29 ± 0.15	1.48 ± 0.04	1.17
*Ss*NRK	1.75 ± 0.07	2.96 ± 0.44	1.0 ± 0.01	0.71

NMN to NAD^+^				
*Ec*NMN-AT	2.5 ± 0.45	1.3 ± 0.12	2.0 ± 0.2	0.85
*Ck*NMN-AT	0.3 ± 0.05	0.4 ± 0.02	10.3 ± 0.02	4.4

cNMN to cNAD^+^				
*Ec*NMN-AT	3.6 ± 0.49	2.8 ± 0.38	1.1 ± 0.1	0.47
*Ck*NMN-AT	0.9 ± 0.03	0.5 ± 0.02	10.4 ± 0.01	4.43

NAD^+^ to NADP^+^				
*Ec*NADK	1.79 ± 0.08	0.51 ± 0.01	6.68 ± 0.17	3.74
*PyH*NADK	0.50 ± 0.05	0.30 ± 0.04	1.21 ± 0.02	0.68

cNAD^+^ to cNADP^+^				
*Ec*NADK	1.56 ± 0.11	0.55 ± 0.22	2.9 ± 0.03	1.67
*PyH*NADK	3.54 ± 0.79	0.40 ± 0.07	0.61 ± 0.06	0.34

Among the NMN-ATs, *Ck*NMN-AT exhibited substantially enhanced turnover, roughly 5-fold higher *k*_cat_ relative to the *Ec*NMN-AT when catalyzing NMN (4.4 *vs.* 0.85 s^−1^), and a 10-fold increase when using cNMN (4.43 *vs.* 0.47 s^−1^), also accompanied by lower *K*_M_ values.

The kinetics for *Ec*NADK and *PyH*NADK with their native substrates have been previously reported;^35,36^ here we have extended the analysis to their performance with cNAD^+^. *Ec*NADK exhibited a ∼5-fold higher *k*_cat_ than *PyH*NADK when catalyzing cNAD^+^ phosphorylation. For the native substrate NAD^+^, *Ec*NADK showed a *K*_M_ of 1.79 ± 0.08 mM and a *k*_cat_ of 3.74 s^−1^, whereas PyHNADK displayed a lower *K*_M_ (0.50 ± 0.05 mM) but a reduced turnover rate (*k*_cat_ = 0.68 s^−1^). While *PyH*NADK displayed a lower *K*_M_ for NAD^+^ compared to *Ec*NADK, its overall catalytic rate remained limited. For cNAD^+^, *Ec*NADK showed a *K*_M_ of 1.56 ± 0.11 mM and a *k*_cat_ of 1.67 s^−1^, while *PyH*NADK displayed a substantially increased *K*_M_ (3.54 ± 0.79 mM) and a lower turnover rate (*k*_cat_ = 0.34 s^−1^). Given that many NAD kinases exhibit narrow substrate specificity, particularly intolerance to modifications at the acceptor (NAD^+^),^37^ both enzymes retained measurable activity with cNAD^+^, highlighting a degree of tolerance toward carbocyclic modification.

Collectively, these data reveal that despite the absence of the canonical ribose moiety, the selected enzymes remain catalytically competent toward carbocyclic substrates, with variable efficiency depending on the phylogenetic origin of the enzyme. To establish the most effective enzyme combinations for coupled assays for NRKs, we first characterized the NMN-ATs, then selected the optimal variant (*Ck*NMN-AT) to drive assays involving the NRKs. However, it is important to consider a kinetic caveat in these coupled assays, ATP is also consumed by *Ck*NMN-AT, which likely results in an overestimation of *K*_M_ values for ATP in the NR-kinase reactions. Here, our focus was primarily on assessing the ability of NRKs to accept the synthetic substrate cNR. The data further provide the first biochemical characterization of *Ss*NRK and *Ck*NMN-AT, confirming their activity with native substrates and extending their substrate scope to include carba analogues. The observation of product/feedback inhibition consistent with native systems further suggests that the fundamental regulatory architecture of NAD^+^ biosynthesis remains conserved in the carbocyclic context.

#### Temperature and pH dependence of enzyme activity

**Fig. 3:**
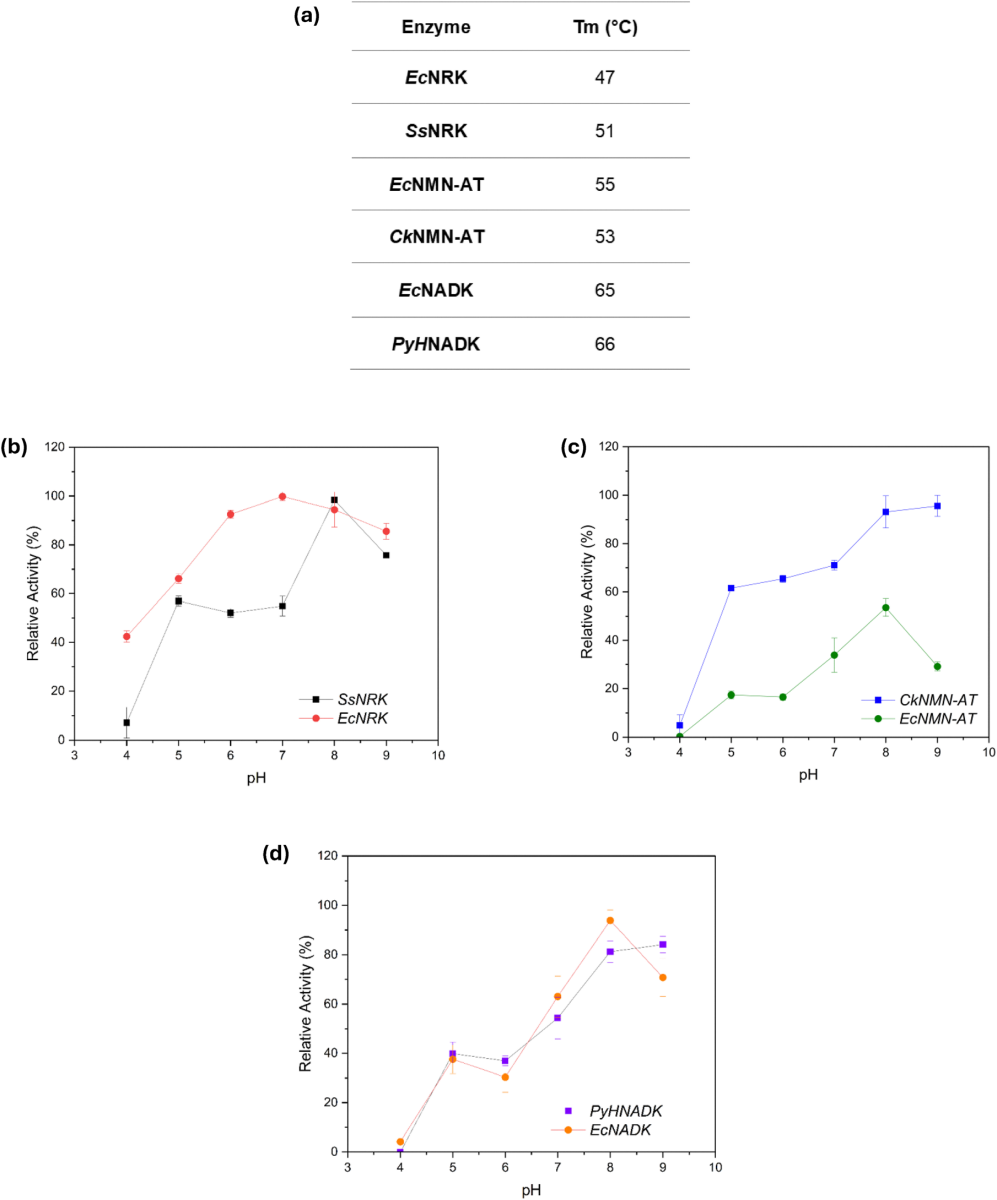
Thermal and pH stability of recombinant enzymes. (a) Summary of melting temperatures (*T*_m_) determined *via* a thermal-shift assay for all recombinant enzymes. *T*_m_ values were derived from the midpoint of the unfolding transitions. (b)–(d) Relative activity profiles of NRKs, NMN-ATs, and NADKs, respectively, across pH 4.0–9.0. Activities were measured under standard assay conditions and normalized to the maximum activity for each enzyme.

To evaluate the suitability of the selected enzymes to temperature and pH conditions before applying them for preparative-scale synthesis, their temperature and pH stability were examined under conditions relevant to biocatalysis. Since overall process efficiency depends on both catalytic performance and operational robustness, these measurements establish a baseline for evaluating enzyme resilience and identifying potential limitations during scale-up or prolonged reactions. Thermal stability was assessed using a fluorescence-based thermal shift assay with SYPRO Orange dye and melting temperatures (*T*_m_) were determined (Fig. 3(a)). Within each enzyme class, comparable thermal profiles were observed, with *T*_m_ values ranging from 47 °C to 66 °C. These temperatures exceed the typical reaction range used for enzymatic synthesis (30–37 °C), indicating sufficient thermal stability for *in vitro* applications. The pH-dependent activity and stability were evaluated across pH 4.0–9.0 (Fig. 3(b)–(d)). All enzymes retained substantial activity between pH 5.0 and 9.0, with optimal activity near pH 8.0, reflecting a preference for slightly alkaline conditions. *Ec*NRK exhibited higher retention of activity than *Ss*NRK across the tested range, while *Ec*NMNAT showed marked sensitivity to acidic conditions. Most enzymes retained 60–100% of their initial activity following incubation in mildly acidic buffers (pH 4.5–6.0), except for *Ec*NMNAT, which lost significant activity. Both NAD^+^ kinase variants (*Ec*NADK and *PyH*NADK) followed similar pH-dependent inactivation trends, maintaining broad activity between pH 6.0 and 9.0.

### Enzymatic synthesis of cNAD^+^

The feasibility of applying the characterized enzymes to the late-stage transformations leading towards cNAD^+^ and cNADP^+^ were evaluated through independent preparative-scale reactions: (i) phosphorylation of carba-nicotinamide riboside (cNR) to carba-nicotinamide mononucleotide (cNMN), (ii) adenylyl transfer of cNMN to carba-NAD^+^, and (iii) phosphorylation of cNAD^+^ to carba-NADP^+^. All reactions were carried out individually at 37 °C in 10 mM HEPES buffer (pH 7.5), with conditions chosen to balance enzymatic compatibility with simplified downstream purification. Enzyme loadings were estimated from the measured kinetic parameters to approximate the catalytic amounts required to reach high substrate turnover within 24 h. Product formation and purity at each stage were verified by chromatographic and spectroscopic analyses.

#### Phosphorylation of cNR by NRK

The *Ec*NRK-catalyzed phosphorylation of cNR afforded cNMN in an isolated molar yield of 56% after 24 h under preparative conditions. Thermodynamic estimates obtained using eQuilibrator indicate that this transformation is strongly favourable under the applied conditions (Δ_r_*G*′° = −33 ± 10 kJ mol^−1^; 
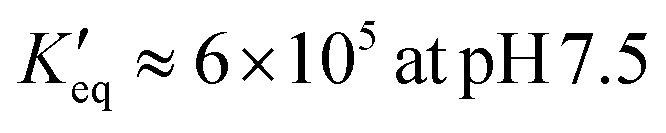
)^5^, indicating that the non-quantitative yield is not imposed by equilibrium constraints. Conversion did not increase further despite the inclusion of an ATP-regeneration system (phosphocreatine/creatine kinase), indicating that ATP availability was not the dominant constraint. Rather, the observed outcome is consistent with a gradual decline in effective catalytic turnover during extended incubation under preparative conditions. Given the known pH sensitivity of *Ec*NRK to reaction environment and the cumulative proton release associated with phosphorylation chemistry, time-dependent changes in reaction composition and nucleotide accumulation provide a plausible explanation for the observed yield ceiling. These effects are, in principle, addressable through standard process refinements such as improved pH control and optimized regeneration capacity.

#### Adenylyl transfer to form cNAD^+^

The adenylylation of cNMN to cNAD^+^ catalyzed by *Ck*NMNAT afforded a 64% molar yield in 10 mM HEPES and (73% when 50 mM HEPES, pH 7.5 was used). Calculations from eQuilibrator (Δ_r_*G*′° = −5.0 ± 5.7 kJ mol^−1^; 
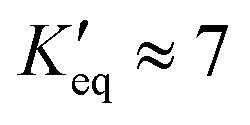
) indicate that this transformation proceeds close to equilibrium under the applied conditions, and the isolated yields closely reflect this intrinsic equilibrium position. Because pyrophosphate (PP_i_) generated during adenylyl transfer was not enzymatically removed, the equilibrium remained unshifted. Incorporation of a PP_i_-hydrolyzing system would therefore be expected to push the reaction further toward product formation and increase the attainable yield.

#### Phosphorylation of cNAD^+^ by NADK

The terminal phosphorylation of cNAD^+^ to cNADP^+^ catalyzed by *Ec*NADK afforded a molar yield of 46% under preparative conditions. Thermodynamic estimates obtained with eQuilibrator (Δ_r_*G*′° = −10.1 ± 5.7 kJ mol^−1^ at pH 7.5, pMg 2.7, *I* = 0.05 M; 
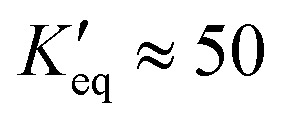
) indicate that the reaction is thermodynamically favourable and not intrinsically equilibrium-limited. The substantially lower observed conversion therefore reflects operational constraints that prevent the system from approaching equilibrium. Progressive product inhibition by cNADP^+^, a well-documented property of NAD kinases, together with time-dependent changes in reaction composition during extended incubation, is likely to limit further turnover. Enhancing ATP regeneration capacity, stabilizing reaction conditions, or implementing *in situ* product removal would be expected to enable higher yields and bring the system closer to its thermodynamic endpoint.

Thermodynamic estimates were derived from eQuilibrator using native NAD^+^-pathway metabolites, as corresponding data for the carbocyclic analogues are unavailable. Because the carba substitution lies remote from the reactive phosphate and adenylate centres, these values provide suitable qualitative proxies for assessing reaction favourability. While ATP regeneration sustained phosphorylation potential, it could not compensate for other limiting factors, including acidification, product inhibition, and intrinsic equilibrium constraints. Incomplete conversion in the kinase-catalyzed steps further reflected limitations of the regeneration module itself: the modest creatine kinase turnover rate and finite phosphocreatine pool (5 mM donor, corresponding to approximately two ATP equivalents per NAD^+^) permitted only partial ATP replenishment, leading to transient ATP depletion and ADP accumulation despite favourable reaction energetics. The moderate yields observed across the enzymatic sequence are therefore consistent with the known regulatory and physicochemical sensitivities of NAD^+^-biosynthetic enzymes. At higher substrate loadings, rapid enzyme precipitation was also observed, particularly upon addition of concentrated ATP or nucleotide intermediates. This behaviour is consistent with the ionic sensitivity of phosphoryl-transfer enzymes, where excess polyanionic substrates can sequester Mg^2+^ and perturb the local charge balance, promoting aggregation under low-ionic-strength conditions.

#### Analytical verification

The identity and integrity of all intermediates and final products were verified using complementary analytical techniques. Thin-layer chromatography (TLC) was used for initial qualitative validation to confirm the purity of intermediates and final products, while LC-ESI-MS and ^1^H NMR spectroscopy provided molecular identity and structural integrity. The mass spectra displayed [M + H]^+^ peaks consistent with the expected monoisotopic masses of cNMN (*m*/*z* 333.0) and cNAD+ (*m*/*z* 662.1). For the NADK-catalyzed phosphorylation to cNADP^+^, the formation of phosphorylated product exhibited an [M + H]^+^ ion at *m*/*z* = 742.1, consistent with the calculated monoisotopic mass of cNADP^+^, thereby supporting successful enzymatic conversion and high product specificity. Further structural validation was achieved through ^1^H (NMR), where the proton spectra displayed chemical shift signatures corresponding to the nicotinamide, adenine, and ribose moieties, with the preservation of expected coupling patterns and integration values. Particularly, downfield shifts in the ribose region and characteristic splitting in the adenosine resonances were consistent with phosphorylation at the 2′-hydroxyl position, confirming the formation of the NADP^+^-type architecture in the modified cofactor. Representative UV chromatograms and mass fragmentation data are shown in Fig. S6 and the corresponding ^1^H NMR spectra are presented in Fig. S7. Collectively, these analyses validate the successful enzymatic preparation of cNAD^+^ and cNADP^+^ with high structural fidelity.

### Synthesis analysis

A direct comparison of the late-stage transformations from cNR to cNAD^+^ highlights distinct differences between chemical and enzymatic approaches in terms of reaction conditions, operational demands, and suitability for biocatalytic applications. Chemically, phosphorylation of cNR to cNMN is achieved using phosphoryl trichloride in trimethyl phosphate, affording high isolated yields (∼88%) but requiring moisture-sensitive reagents, low-temperature control, and harsh activation chemistry. In contrast, the NRK-catalyzed phosphorylation proceeds in aqueous buffer at 37 °C and neutral pH towards cNMN in 56% yield under mild conditions without chemical activation, albeit with sensitivity to reaction composition and enzyme stability over extended incubation. Similarly, chemical conversion of cNMN to cNAD^+^ relies on activated adenosyl donors and metal-assisted diphosphate coupling in formamide at elevated temperatures, providing cNAD^+^ in 61% yield. The enzymatic adenylylation catalyzed by NMN-AT operated under aqueous, near-physiological conditions and reached up to 73% yield, while avoiding protecting groups and reactive intermediates, but remained constrained by intrinsic equilibrium limitations in the absence of pyrophosphate hydrolysis.

Beyond reaction performance, differences in downstream processing become apparent across the cNR → cNMN → cNAD^+^ sequence. In the chemical route, both phosphorylation and subsequent diphosphate coupling require removal of reactive phosphorylating agents or activated nucleotide intermediates, residual metal salts, and side products, typically involving aqueous work-up followed by ion-exchange chromatography under carefully controlled conditions. In contrast, the enzymatic cascade is conducted entirely in aqueous buffer and allows direct processing of the reaction mixture after enzyme removal *via* ultrafiltration. Purification is then performed in a product-specific manner, primarily by ion-exchange chromatography, where residual nucleotides, salts, and cofactors are separated under comparatively mild elution conditions. Final isolation is achieved by freeze-drying without intermediate purification steps between transformations.

From a process perspective, the chemical route offers robustness and consistent fair yields, whereas the enzymatic route emphasizes operational simplicity, selectivity, and compatibility with cell-free biocatalytic systems, albeit with yields that depend sensitively on enzyme performance and reaction control. A defining advantage of the enzymatic strategy is its extension beyond cNAD^+^ to cNADP^+^*via* NAD kinase catalysis, a transformation that is challenging *via* chemical means and has rarely been reported.

### Thermal stability of cNAD^+^

**Fig. 4:**
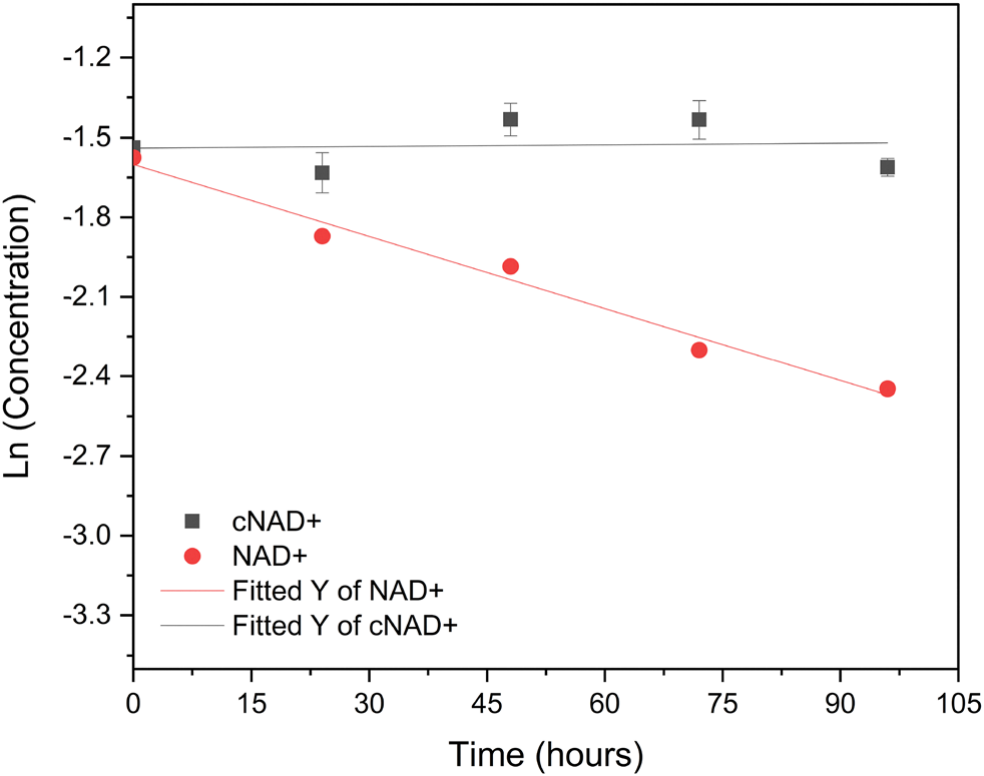
Thermal degradation kinetics of NAD^+^ and cNAD^+^ at 50 °C. First-order kinetic fits of ln(*C*/*C*_0_) *versus* time used to calculate decay rate constants (*k*_*i*_) and half-lives (*t*_1/2_). NAD^+^ undergoes rapid degradation, whereas cNAD^+^ remains essentially stable under identical conditions.

To extend previous findings on cNADP^+^ stability reported by Zachos *et al.*,^13^ and examine whether analogous thermal stabilization is conferred upon cNAD^+^, we assessed the thermostability of cNAD^+^ relative to NAD^+^ by comparing their half-lives and first-order decay rate constants (*k*_*i*_). Equimolar solutions of both cofactors were incubated at 50 °C and monitored by HPLC over 96 h. For both cofactors, decay rate constants (*k*_*i*_) were determined from first-order degradation kinetics by plotting ln(*C*/*C*_0_) *versus* time (Fig. 4). Under these conditions, NAD^+^ degraded at a rate of *k*_*i*_ = 0.0091 mM h^−1^ (*t*_1/2_ = 76 h), whereas cNAD^+^ remained essentially unchanged (*k*_*i*_ = 0.0005 mM h^−1^, *t*_1/2_ > 1386 h). After 96 h at 50 °C, NAD^+^ showed a pronounced decrease in peak intensity, while cNAD^+^ retained its initial concentration. Consistent with this interpretation, HPLC chromatograms revealed multiple degradation products for NAD^+^ but none for cNAD^+^ (Fig. S4). This marked difference mirrors the stability previously reported for cNADP^+^ and confirms that the carbocyclic substitution substantially enhances cofactor robustness.

### Enzyme acceptance of cNAD^+^ by oxidoreductases

**Fig. 5:**
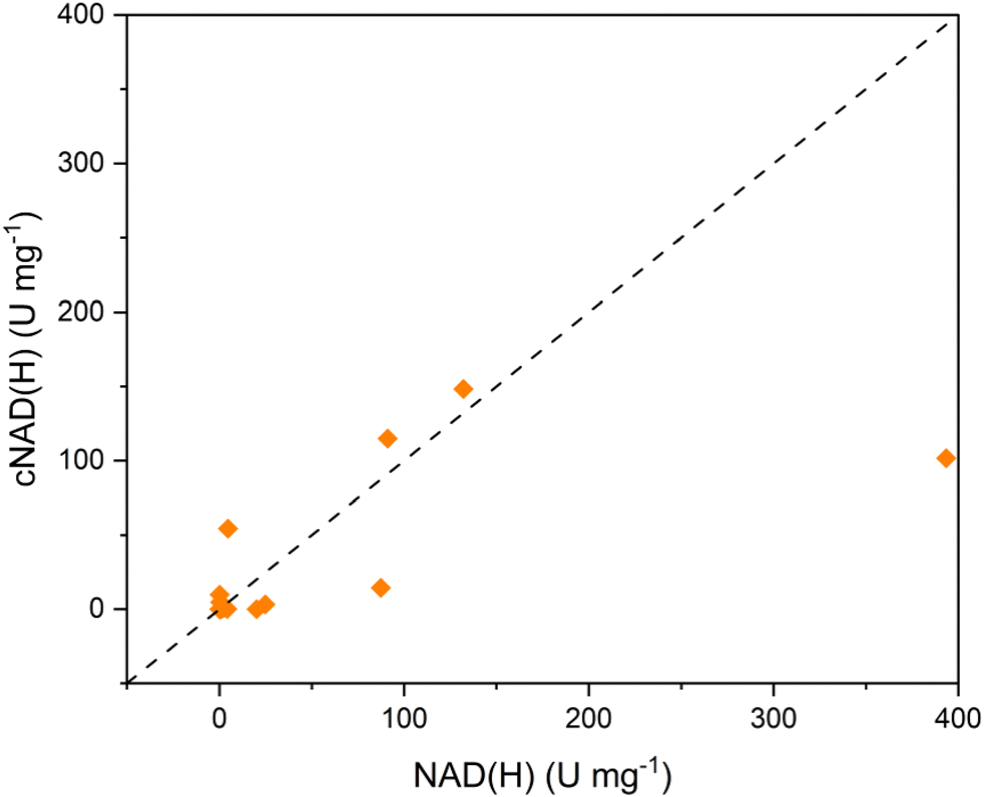
Comparison of oxidoreductase activity with native NAD(H) and carbocyclic cNAD(H). Specific activities (U mg^−1^) measured for oxidoreductases using NAD(H) are plotted against the corresponding activities obtained with cNAD(H) under identical assay conditions. Each data point represents one enzyme–substrate pair (see Table S1). The dashed line indicates parity (*y* = *x*). A general positive correlation is observed, demonstrating that cNAD(H) is broadly accepted as a redox cofactor, with enzyme-dependent variations in activity.

Furthermore, to confirm that the carbocyclic dinucleotide remains functionally competent as a redox cofactor, we evaluated its acceptance by a representative panel of NAD^+^-dependent oxidoreductases under standard assay conditions. A total of 23 enzymes of different subclasses commonly used in biocatalysis were examined with their native substrates (Table S1). In line with our previous findings for cNADP(H),^13^ most enzymes retained measurable activity with cNAD(H). Specific activities spanned several orders of magnitude and showed a clear positive correlation with those obtained using NAD(H) Fig. 5, indicating that carbocyclic substitution does not disrupt the fundamental hydride-transfer chemistry. While individual enzymes displayed reduced activity relative to the native cofactor, no systematic loss of function was observed, confirming that cNAD^+^ is broadly compatible with diverse oxidoreductase classes. Together, these results extend prior observations from cNADP^+^ to cNAD^+^ and support its utility as a stable, non-natural redox cofactor for biocatalytic applications.

## Conclusions

In this study, we present complementary chemical and enzymatic strategies for the preparation and evaluation of carbocyclic nicotinamide cofactors. A stereoselective chemical route provides reliable access to cNAD^+^, while enzymatic late-stage transformations demonstrate that native NAD^+^-biosynthetic enzymes can accommodate carbocyclic substrates and enable access to cNMN, cNAD^+^, and, notably, cNADP^+^. Although the enzymatic synthesis is influenced by factors such as product inhibition and reaction-environment stability during extended turnover, these limitations can be addressed through established process controls, including optimized cofactor regeneration and product removal strategies. In addition, cNAD^+^ exhibits markedly enhanced thermal stability relative to NAD^+^ and functions as a competent redox cofactor for a broad range of NAD^+^-dependent oxidoreductases. Together, these findings establish a practical chemoenzymatic framework for accessing robust, non-natural nicotinamide cofactors and support their further development for applications in biocatalysis, cofactor engineering, and cell-free synthetic systems.

## Author contributions

Z. D., J. J. F., and V. S. conceived and designed the study. Z. D. carried out enzymatic synthesis, biochemical characterization, kinetic analyses, cofactor thermostability studies, and data analysis. J. J. F. performed the chemical synthesis and analytical characterization of carbocyclic cofactors. M. D. conducted oxidoreductase screening and enzymatic activity assays. N. R. T. cloned and expressed the enzymes used in the enzymatic synthesis studies. L. S. contributed with technical advice, conceptual input, and project administration. Z. D. wrote the original manuscript. Z. D. and V. S. reviewed and edited the manuscript.

## Conflicts of interest

The authors declare no conflicts of interest.

## Supplementary Material

CB-OLF-D6CB00036C-s001

## Data Availability

The data supporting this study have been included as part of the supplementary information (SI). Supplementary information: experimental procedures, supporting data and supplementary references. See DOI: https://doi.org/10.1039/d6cb00036c.
