# Supplementary material for: Synthesis of thermostable artificial nicotinamide cofactors: carba-NAD+ and carba-NADP+
Source: RSC Chem Biol. 2026 May 5;7(7):1330–40. doi: 10.1039/d6cb00036c (PMC13185757; doi:10.1039/d6cb00036c)
Supplement: CB-007-D6CB00036C-s001 [file CB-007-D6CB00036C-s001.pdf]

Supplementary Information (SI) for RSC Chemical Biology.
This journal is © The Royal Society of Chemistry 2026

Supplementary Information

Synthesis of Thermostable Artificial Nicotinamide Cofactors: Carba-NAD$^+$ and Carba-NADP$^+$

Zinnia Dsouza, Jan-Simon Jeshua Friedrichs, Manuel Döring, Nicolas Rincón Téllez, Luca Schmermund, Volker Sieber

Contents

1. Experimental Procedures 2
   1.1 Chemical Synthesis 2
   1.2 Cloning, Expression, and Purification 9
   1.3 Enzyme Characterization 11
   1.4 Preparative-Scale Enzymatic Synthesis 14
   1.5 Thermostability anaylsis : NAD$^+$ and cNAD$^+$ 19
   1.6 Oxidoreductase Activity Assays with NAD(H) and cNAD(H) 20
2. Supplementary Figures 22
3. NMR spectra 34
4. Supplementary Table 45
5. Supplementary References 48

1. Experimental Procedures

1.1 Chemical Synthesis

(1R,4S,5R,6S)-5,6-Dihydroxy-2-azabicyclo[2.2.1]heptan-3-one (1)

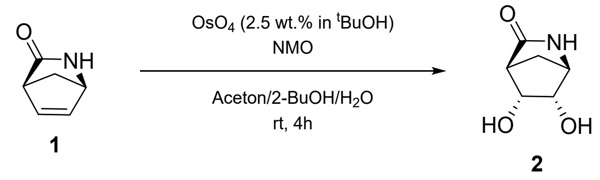

A 350 mL pressure tube was charged with 25.0 g (1R,4S)-2-azabicyclo[2.2.1]hept-5-en-3-one **1** (1.00 eq., 229 mmol) and 40.3 g *N*-methylmorpholine *N*-oxide (1.50 eq., 344 mmol). Both compounds were suspended in a mixture of 70.0 mL acetone, 70.0 mL 2-butanol and 70.0 mL H_2O before addition of 18.0 mL of 2.5% (w/w) OsO_4 in *tert*-butanol. Then, the tube was sealed with a PTFE cap and the reaction mixture stirred at 70 °C for 4 h. Subsequently, $NaHSO_3$ (2.50 g) were added and the mixture stirred for additional 45 minutes while cooling down to ambient temperature. The reaction mixture was filtered and the solvent removed under reduced pressure. The residue was purified by DCVC (10.0x5.00 cm, dry load) using a solvent gradient starting from 100% EtOAc to a mixture of EtOAc/2-propanol (75:25%). The product precipitated while concentrating the eluent under reduced pressure, resulting in a first fraction of pure product. The filtrate was further concentrated under reduced pressure and recrystallized from EtOH yielded a second pure product fraction. Compound **2** was obtained as amorphous, white substance (24.1 g, 168 mmol, 73% yield).

R_f: 0.24 (20% 2-propanol in EtOAc, $KMnO_4$ staining).

^1H NMR (400 MHz, DMSO-D_6) δ 7.52 (s, 1H), 4.98 (s, 2H), 3.77 (dd, *J* = 6.0, 1.6 Hz, 1H), 3.70 (dd, *J* = 6.0, 1.6 Hz, 1H), 3.43 (s, 1H), 2.25 (s, 1H), 1.89 (d, *J* = 9.7 Hz, 1H), 1.72 (dt, *J* = 9.9, 1.8 Hz, 1H).

***tert*-Butyl (3aS,4S,7S,7aR)-2,2-dimethyl-6-oxotetrahydro-4,7-methano[1,3]dioxolo[4,5-c]pyridine-5(4H)-carboxylate**

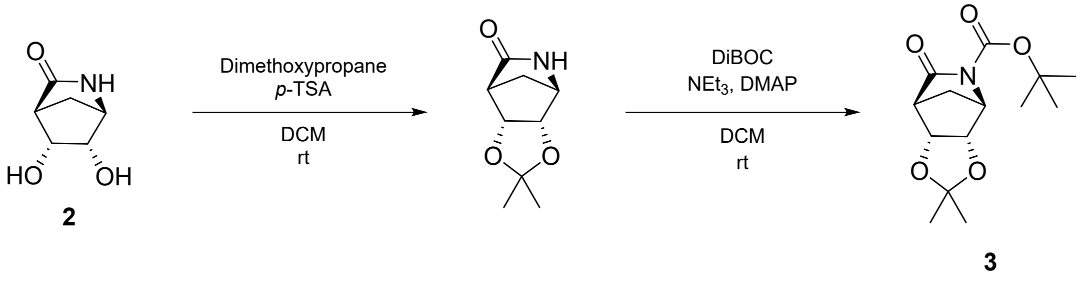

In a 250 mL round-bottom flask, 10.0 g of compound **2** (1.00 eq., 69.9 mmol) and 17.1 mL dimethoxypropane (2.00 eq., 14.5 g, 140 mmol) were dissolved in 100 mL DCM. Subsequently, 0.66 g *p*-toluenesulfonic acid monohydrate (0.05 eq., 3.49 mmol) was added and the mixture stirred at ambient temperature for 24 h. After the reaction was finished, the mixture was diluted with DCM and washed with a 0.05 M sodium hydroxide solution followed by brine. The aqueous phase was then extracted DCM (3x100 mL). The combined organic phases were dried over sodium sulfate, filtered and the solvent removed under reduced pressure. The crude product (12.6 g, 68.9 mmol, 99% yield) was used without further purification in the next synthesis step.

^{1}H NMR (400 MHz, CHLOROFORM-*D*) δ 6.34 (s, 1H), 4.53 (d, *J* = 5.5 Hz, 1H), 4.40 (d, *J* = 5.5 Hz, 1H), 3.78 (s, 1H), 2.71 (s, 1H), 2.15 – 2.10 (m, 1H), 2.07 – 2.01 (m, 1H), 1.47 (s, 3H), 1.34 (s, 3H).

For the next step, 12.0 g of crude protected lactam (1.00 eq., 65.5 mmol) were dissolved in 200 mL anhydrous DCM. Under a nitrogen atmosphere, 9.48 mL anhydrous triethylamine (1.10 eq., 72.1 mmol) were added followed by dropwise addition of 18.1 mL di-*tert*-butyldicarbonate (1.20 eq., 78.6 mmol) in 40.0 mL anhydrous DCM. The reaction mixture was stirred for 4 h at room temperature. Then, the mixture was diluted with DCM and washed with a 0.3 M $KHSO_4$ solution and brine. The aqueous phase was extracted thrice with DCM and the

combined organic phases dried over Na_2SO_4. The suspension was filtered and the solvent removed under reduced pressure. The crude material was recrystallized from *n*-hexane/ EtOH (100:20 mL) to obtain a first fraction (14.2 g). The mother liquor was concentrated under reduced pressure and the residue was purified by DCVC (6.00 x 3.00 cm, cyclohexane (CH) /EtOAc; 10:1 to 9:1, 8:1, 7:1, 6:1, 5:1) yielding a second fraction (2.70 g). The product **3** was obtained as white amorphous solid in a total yield of 91% (16.9 g, 59.6 mmol).

R_f: 0.41 (CH/EtOAc 7:3)

^{1}H NMR (400 MHz, CHLOROFORM-*D*) δ 4.58 (d, *J* = 5.4 Hz, 1H), 4.47 (d, *J* = 5.5 Hz, 1H), 4.42 (s, 1H), 2.09 (dt, *J* = 10.9, 1.7 Hz, 2H), 1.98 (dt, *J* = 10.9, 1.6 Hz, 2H), 1.51 (s, 14H), 1.48 (s, 5H), 1.35 (s, 5H).

tert-butyl ((3aS,4R,6R,6aR)-6-(hydroxymethyl)-2,2-dimethyltetrahydro-4H-cyclopenta[d][1,3]dioxol-4-yl)carbamate

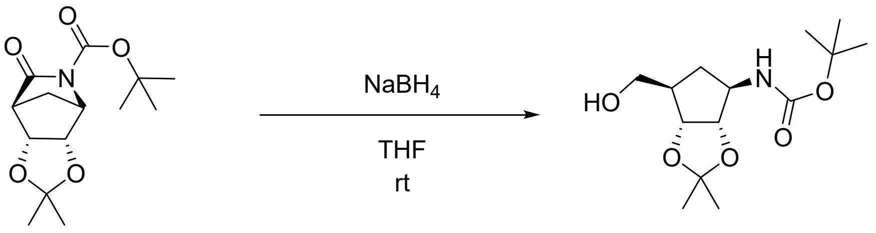

3 4

To a stirred solution of 12.5 g **3** (1.00 eq., 44.1 mmol) in 500 mL dry THF, 6.68 g $NaBH_4$ (4.00 eq., 176 mmol) was added in small portions and the mixture stirred under a nitrogen atmosphere for 24h. After reaction was finished, the mixture was cooled to 0°C with an ice-bath and unreacted $NaBH_4$ was quenched using a saturated NH_4Cl solution. Then, the THF phase was diluted with EtOAc and washed with brine. The combined aqueous phases were extracted thrice with EtOAc. The combined organic phases were dried over Na_2SO_4, filtered and the solvent removed under

reduced pressure. The crude material was purified by DCVC (10.0 x 5.00 cm) using a solvent gradient starting from 5% EtOAc in CH to 50% EtOAc in CH. The product **4** was obtained as sticky, colorless oil that crystalizes in 84% yield (10.7 g, 37.2 mmol).

R_f: 0.21 (CH/EtOAc 1:1)

^{1}H NMR (400 MHz, DMSO-D_6) δ 6.91 (d, J = 7.7 Hz, 1H), 4.76 (t, J = 5.0 Hz, 1H), 4.35 – 4.30 (m, 1H), 4.29 – 4.22 (m, 1H), 3.76 – 3.65 (m, 1H), 3.44 – 3.34 (m, 2H), 2.12 – 2.00 (m, 1H), 1.37 (s, 9H), 1.37 (s, 3H), 1.19 (s, 3H).

(1R,2S,3R,5R)-3-amino-5-(hydroxymethyl)cyclopentane-1,2-diol

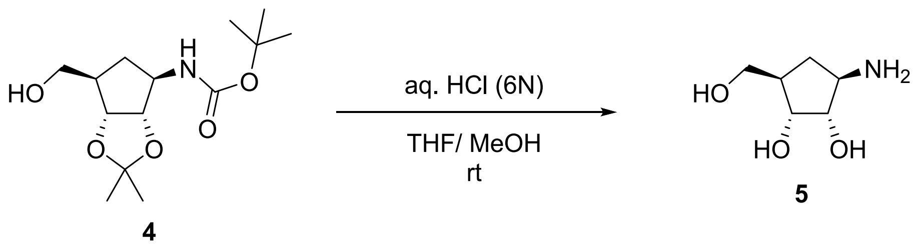

In a 1000 mL three-necked round-bottom flask, 10.4 g **4** (1.00 eq, 36.2 mmol) were dissolved in 400 mL of a THF/ MeOH (1:1) mixture. Via a dropping funnel, 100 mL 6M HCl are carefully and drop wisely added. After the addition was finished, the mixture was stirred at ambient temperature for 24h. Then, the reaction mixture was concentrated under reduced pressure. The residue is diluted with H_2O and washed with DCM. The organic phase was washed twice with H_2O. The combined aqueous phase were concentrated under reduced pressure and purified using a cation exchanger (Dowex 50W X8, elution with 1.5% NH_4OH). The product **5** was obtained as transparent, light brownish oil with a yield of 98% (5.21 g, 35.4 mmol).

^{1}H NMR (400 MHz, D_2O) δ 3.91 – 3.82 (m, 1H), 3.68 – 3.52 (m, 3H), 3.22 – 3.10 (m, 1H), 2.23 – 2.13 (m, 1H), 2.12 – 2.02 (m, 1H), 1.07 – 0.94 (m, 1H).

3-Carbamoyl-1-((1R,2S,3R,4R)-2,3-dihydroxy-4-(hydroxymethyl)cyclopentyl)pyridin-1-ium chloride (Zincke-Reaction)

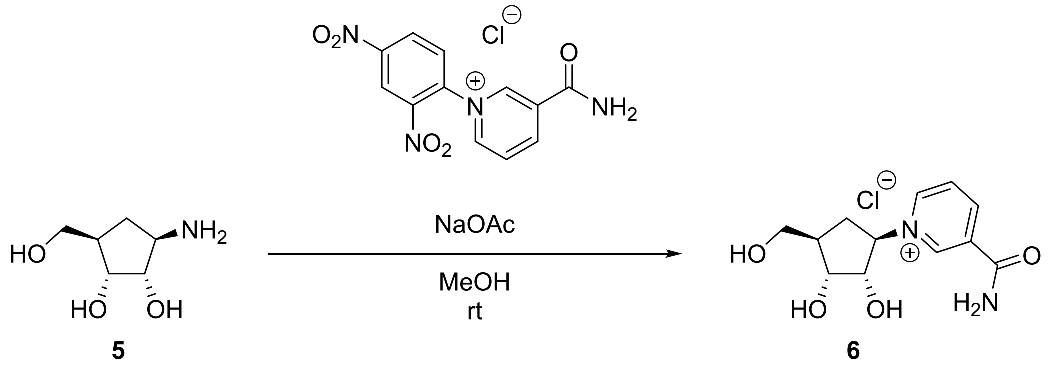

To a solution of 4.35 g **5** (1.03 eq., 29.6 mmol) in 20 mL MeOH, a solution of 9.32 g Zincke reagent (1.00 eq., 28.7 mmol) in 20 mL MeOH was added. The mixture immediately turned red violet. Next, 2.55 g NaOAc (1.05 eq, 31.1 mmol) were added, and the reaction stirred at ambient temperature for 2h. To remove excess Zincke reagent, 1.50 mL ammonia (32%) were added and the reaction mixture stirred for additional 10 minutes. Subsequently, the solvent was removed under reduced pressure and the residue suspended in H_2O to precipitate 2,4-dinitroanilin. The solid material was filtered off and the filtrate concentrated under reduced pressure. The residue was dissolved in MeOH and purified by DCVC (10.0 x 5.00 cm) using a solvent gradient from 5% B (B = 5% HOAc in MeOH) in EtOAc to 100% B. To remove any residual HOAc in the product, the material was further purified using anion exchange chromatography (Dowex 1x8, Chloride form, elution with H_2O). The product **6** was obtained in 76% yield (6.50 g, 22.5 mmol) as bright orange foam after drying under high vacuum at elevated temperature (40 – 45°C).

^{1}H NMR (400 MHz, D_2O) δ 9.38 (d, *J* = 1.7 Hz, 1H), 9.13 (d, *J* = 6.1 Hz, 1H), 8.92 (d, *J* = 8.2 Hz, 1H), 8.22 (dd, *J* = 8.1, 6.2 Hz, 1H), 5.06 (ddd, *J* = 11.1, 9.5, 7.8 Hz, 1H), 4.41 (dd, *J* = 9.5, 5.9 Hz, 1H), 4.08 (dd, *J* = 5.9, 3.0 Hz, 1H), 3.72 (d, *J* = 6.0 Hz, 2H), 2.67 (dt, *J* = 13.2, 8.2 Hz, 1H), 2.37 – 2.25 (m, 1H), 2.03 – 1.90 (m, 1H).

3-Carbamoyl-1-(2,4-dinitrophenyl)pyridine-1-ium chloride (Zincke reagent)

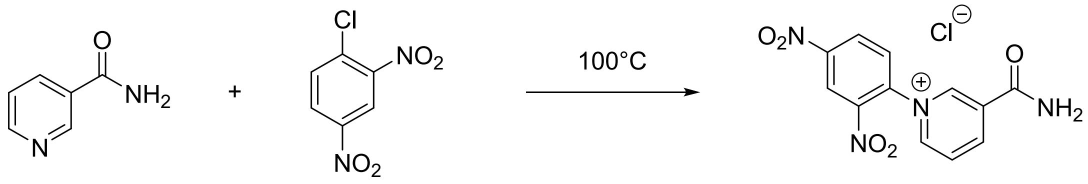

A 250 mL round-bottom flask was charged with 15.4 g Chloro-2,4-dinitrobenzene (1.00 eq., 75.0 mmol) and 9.16 g nicotinamide (1.00 eq., 75.0 mmol). Before mixing, both compounds were finely powdered. Then, the mixture was melted at 100°C and further stirred for about 20 minutes. After the mixture cooled to ambient temperature, it was dissolved in MeOH and mixed with silica. The solvent was removed under reduced pressure and the dry load used for purification via DCVC (10.0 x 5.00 cm). The product was eluted using a griednt of 20% MeOH in DCM to 40% MeOH in DCM. The product containing fraction after the first purification step was concentrated under reduced pressure and the residue mixed with H_2O. While mixing with water, a brown solid precipitated. The solid was filtered off and the filtrate further purified by anion-exchange chromatography (Dowex 1x8, elution with H_2O). The product was obtained as bright orange foam in 61% yield (14.9 g, 46.0 mmol).

^{1}H NMR (400 MHz, D_2O) δ 9.73 (s, 1H), 9.43 (d, J = 2.6 Hz, 1H), 9.41 (s, 1H), 9.34 (d, J = 7.8 Hz, 1H), 9.00 (d, J = 8.7 Hz, 1H), 8.56 (dd, J = 8.5, 6.0 Hz, 1H), 8.34 (d, J = 9.0 Hz, 1H).

((1R,2R,3S,4R)-4-(3-carbamoylpyridin-1-ium-1-yl)-2,3-dihydroxycyclopentyl)methyl hydrogen phosphate

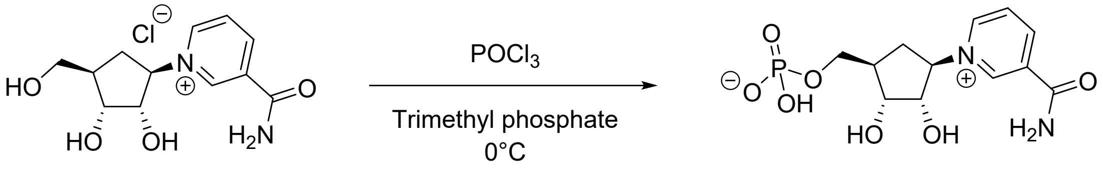

6 **7**

In a 100 mL Schlenk flask, 3.00 g **6** (1.00 eq., 9.87 mmol) is suspended in 25.0 mL $PO(OMe)_3$. The mixture is evacuated for about 2 minutes before backfilling the vacuum with N_2. The mixture is cooled to 0°C followed by the dropwise addition of 2.76 mL $POCl_3$ (3.00 eq., 29.6 mmol) under N_2 atmosphere. The mixture is stirred for 1h at 0°C before quenching excess $POCl_3$ with 25 mL H_2O. The mixture is heated up to ambient temperature and further diluted with 100 mL H_2O. The aqueous solution was applied to a cation exchange resin (Dowex 50W X8, H^+-form, elution with H_2O) for purification. The solvent was removed under reduced pressure and the residue dissolved in a small volume of H_2O. The pure product **7** was obtained as yellowish foam, after freeze-drying, in 88% yield (2.89 g, 8.69 mmol).

^1H NMR (400 MHz, D_2O) δ 9.27 (s, 1H), 9.06 (d, *J* = 6.0 Hz, 1H), 8.81 (d, *J* = 8.7 Hz, 1H), 8.13 (t, *J* = 6.3 Hz, 1H), 5.03 – 4.91 (m, 1H), 4.44 – 4.32 (m, 1H), 4.11 – 4.03 (m, 1H), 3.98 – 3.90 (m, 1H), 3.90 – 3.77 (m, 1H), 2.68 – 2.56 (m, 1H), 2.39 – 2.27 (m, 1H), 2.07 – 1.94 (m, 1H).

carba-nicotinamide adenine dinucleotide (cNAD$^+$)

CarbaNMN (cNMN) was converted to cNAD$^+$ via diphosphate coupling using adenosine 5′-monophosphomorpholidate as the adenosyl transfer reagent. To a solution of cNMN in formamide, manganese(II) chloride was added, followed by adenosine 5′-monophosphomorpholidate 4-morpholine-N,N′-dicyclohexylcarboxamidine salt (**12**). The reaction mixture was stirred at room

temperature for 24 h. After completion, the reaction mixture was subjected to ion-exchange chromatography using DOWEX 1×8 resin (formate form). The product was eluted under mild conditions with 0.03 M formic acid, affording $cNAD^+$ as the desired product in 61% isolated yield. Adenosine 5′-monophosphomorpholidate (CAS 24558-92-7) was purchased from Sigma-Aldrich and used without further purification.

1.2 Cloning, Expression, and Purification

Codon-optimized synthetic genes encoding the selected enzyme candidates were obtained as GeneArt Strings DNA fragments (Thermo Fisher Scientific, Germany) for heterologous expression in Escherichia coli. Genes were cloned into the pET-28a (+) expression vector, which confers a kanamycin resistance marker, using Gibson Assembly. A total of 10 µL of the ligation mixture was used to transform chemically competent E. coli DH5α (Invitrogen, Thermo Fisher Scientific) cells via heat shock. Positive clones were screened, and the correct insertion of target genes into the pET-28a backbone was confirmed by Sanger sequencing (Eurofins Genomics, Germany).

For protein expression, the validated plasmids were used to transform the *E. coli* BL21(DE3) Star (Invitrogen, Thermo Fisher Scientific) competent cells. Single colonies were used to inoculate LB medium (10 mL in 100 mL baffled flasks) containing 100 µg/mL kanamycin and cultured overnight at 37 °C and 140 rpm. The pre-culture was used to inoculate 1 L of ZYM-5052 autoinduction medium supplemented with 100 µg/mL kanamycin. Cultures were grown at 37 °C and 110 rpm until reaching an optical density at 600 nm (OD_{600}) of 0.6. The incubation temperature was reduced to 20 °C, and the protein expression proceeded for 20 h.

Cells were harvested by centrifugation at 4 °C and 4,500 rpm for 20 minutes. The supernatant was discarded, and the cell pellets were stored at –80 °C until further processing. For lysis, cell pellets were resuspended in 40 mL of binding buffer (20 mM HEPES, pH 7.5, 20 mM imidazole, 500 mM NaCl, 5% w/v glycerol) in 50 mL Falcon tubes. DNase I (5 µg/mL; AppliChem) and 2 mM $MgCl_2$ were added to the suspension. Cells were disrupted by sonication on ice (20 minutes total time; amplitude 70%; 0.5 duty cycle). Cell debris was removed by centrifugation at 35,000 × g for 30 minutes at 4 °C. The resulting supernatant was filtered through a 0.45 µm cellulose membrane (VWR, Germany) and applied to a His GraviTrap™ affinity column (Merck Millipore) for purification of His-tagged proteins.

Proteins were eluted using an elution buffer containing 50 mM HEPES (pH 7.5), 500 mM imidazole, 500 mM NaCl, and 5% w/v glycerol. Buffer exchange was subsequently performed using PD-10 desalting columns (Merck Millipore) with desalting buffer composed of 100 mM HEPES (pH 7.5), 150 mM NaCl, and 5% w/v glycerol. Purified enzymes were flash-frozen in liquid nitrogen and stored at –80 °C for downstream applications. Protein purity and subunit molecular weight were assessed using SDS-PAGE.

The native molecular mass of each enzyme was determined by size-exclusion chromatography on a Superdex 200 10/300 GL column (GE Healthcare), pre-equilibrated with 50 mM potassium phosphate buffer (pH 7.2) containing 0.15 M NaCl. A gel filtration standard kit (Cytiva) was used to calibrate the column. The partition coefficient (K_{av}) for each protein was calculated using Equation 1:

$$K_{av} = (Ve - Vo) / (Vc - Vo)$$

Where Ve is the elution volume of the protein, Vo is the void volume, and Vc is the column volume. K_{av} values were plotted against the logarithm (base 10) of the molecular weights of the standard proteins to generate a calibration curve.

1.3 Enzyme Characterization

Chemicals and Substrate Preparation

Nicotinamide riboside (NR) was obtained from Cayman Chemical, and nicotinamide mononucleotide (NMN) was purchased from Merck. NAD^+ was purchased from Roche, while ATP, D-glucose, D-glucose-6-phosphate, and magnesium chloride hexahydrate were purchased from Carl Roth (Germany). Glucose-6-phosphate dehydrogenase ($NADP^+$-dependent) was purchased from Thermo Fisher Scientific. *Sulfolobus solfataricus* glucose dehydrogenase (*Ss*GDH; UniProt ID: O93715) was prepared in-house following established protocols[1]. Creatine Phosphate and creatine kinase were purchased from Sigma Aldrich.

Carba-substituted substrates: carba-nicotinamide riboside (cNR), carba-nicotinamide mononucleotide (cNMN), and carba-NAD^+ ($cNAD^+$) as substrates for enzymatic validation were synthesized chemically using adapted and modified procedures based on previously reported methods[2-4] .

Spectrophotometric assays:

a) Nicotinamide Ribosyl kinase (NRK)

NRK activity was quantified using a coupled spectrophotometric assay adapted and modified from previously reported methods [5]. In this assay, conversion of nicotinamide riboside (NR) or its carba mimic (cNR) to the corresponding mononucleotide (NMN or cNMN) was indirectly monitored via NADH or cNADH formation through a two-enzyme cascade. Specifically, NMN or cNMN was adenylylated to NAD^+ or $cNAD^+$ by *C. koseri* NMN adenylyltransferase (*Ck*NMN-AT), followed by reduction to NADH or cNADH through *S. solfataricus* glucose dehydrogenase (*Ss*GDH)-catalyzed oxidation of D-glucose. Reactions contained 50 mM HEPES buffer (pH 7.5), 10 mM $MgCl_2$, 10 mM D-glucose, 1mg/mL *Ck*NMN-AT and *Ss*GDH, and varying concentrations of substrates NR / cNR and ATP.

b) Nicotinamide mononucleotide adenylyl transferase (NMN-AT)

NMN-AT activity was assessed via a continuous spectrophotometric assay adapted and modified from previously published protocols[6]. This assay couples NAD^+ or $cNAD^+$ formation from NMN or cNMN to NADH or cNADH production via glucose dehydrogenase. Standard reactions contained 50 mM HEPES buffer (pH 7.5), 10 mM $MgCl_2$, 10 mM D-glucose, 1mg/mL *Ss*GDH, and varying concentrations of substrates NMN/cNMN and ATP. Negative control experiments confirmed that *Ss*GDH does not act on (c)NR, (c)NMN, and no NADH formation was observed in the absence of substrate.

c) NAD^+ Kinase

NAD^+ kinase activity was evaluated using a two-step coupled assay[7], in which the formation of $NADP^+$ or $cNADP^+$ was quantified indirectly via glucose-6-phosphate dehydrogenase (G6PDH)-mediated production of NADPH. Reaction mixtures consisted

of 50 mM HEPES buffer (pH 7.5), 10 mM $MgCl_2$, 10 mM glucose-6-phosphate, 0.5 U G6PDH, and varying concentrations of substrates of NAD^+/$cNAD^+$ and ATP.

General Conditions and Data Analysis

All absorbance-based measurements were performed in 96-well microplates (Greiner Bio-One, polystyrene, F-bottom, clear) using an Epoch 2 microplate reader (BioTek Instruments) at 37 °C. NADH formation was monitored at 340 nm, while cNADH formation was recorded at 360 nm (Supplementary Figure S5). Reactions were initiated by the addition of 10 µL of appropriately diluted enzyme to 90 µL of reaction mixture (Total Volume: 100 µL). Initial reaction velocities were derived from the linear region of the absorbance curves and analyzed using the Michaelis–Menten model. Data analysis was performed with OriginPro (v2021b, OriginLab).

Thermal Stability Determination by Thermofluor Assay

Protein thermal stability was assessed using a SYPRO Orange-based Thermofluor assay. Reactions (25 µL total volume) contained 2 µL of SYPRO Orange dye (1:80 dilution, Invitrogen), 2 µL of protein solution (1 mg mL^1), and 20 mM HEPES buffer (pH 7.5). Assays were conducted on a CFX96 Touch Real-Time PCR Detection System (Bio-Rad, USA) with temperature ramping from 4 °C to 100 °C in 1 °C increments, with a 1-minute equilibration per step. Fluorescence signals were recorded as a function of temperature and melting temperatures (Tm) were calculated from the minima of the first derivative (–dF/dT) of the fluorescence curve. Data analysis and curve fitting were performed using CFX Maestro software, following the manufacturer's Protein Thermal Shift protocol (Bio-Rad).

pH Stability Assay

The pH stability of the enzyme was evaluated by incubating enzyme aliquots in a series of buffers spanning pH 4.0 to 9.0, followed by measurement of residual activity under standard assay conditions. To ensure consistent ionic strength, all buffers were prepared at a final concentration of 50 mM. The following buffer systems were employed: sodium acetate (pH 4.0 and 5.0), potassium phosphate (pH 6.0 and 7.0), and Tris-HCl (pH 8.0 and 9.0). All buffers were freshly prepared and adjusted to the desired pH at room temperature using either HCl or NaOH.

Enzyme samples were diluted into the respective pH buffers and incubated on ice for 6 h to minimize thermal denaturation during exposure. Post-incubation, samples were buffer-exchanged into assay buffer (50 mM HEPES, pH 7.0) to restore neutral conditions prior to activity measurements. Enzyme concentration was verified after buffer exchange to ensure consistency across all samples.

Residual enzymatic activity was assayed at 37 °C under standard substrate conditions. Activities were normalized to that of a control sample maintained at pH 7.0 without prior incubation, which was defined as 100% relative activity. The residual activity at each pH was expressed as a percentage of this reference value, and the resulting data were plotted to generate the enzyme's pH stability profile.

1.4 Preparative-Scale Enzymatic Synthesis

Synthesis of Carba-Nicotinamide Mononucleotide (cNMN) from Carba-Nicotinamide Riboside (cNR)

Carba-nicotinamide mononucleotide (cNMN) was synthesized via ATP-dependent phosphorylation of 5 mM carba-nicotinamide riboside (cNR). The reaction contained 2.5 mM adenosine triphosphate (ATP), 10 mM magnesium chloride ($MgCl_2$). To maintain ATP levels throughout the reaction, an ATP regeneration system based on creatine phosphate and creatine phosphokinase was employed, using 5 mM of creatine phosphate and 1 mg of commercial creatine phosphokinase. The phosphorylation of cNR to cNMN was catalyzed by 0.42 U of *Ec*NRK.

Synthesis of $cNAD^+$ from cNMN

Carba-nicotinamide adenine dinucleotide ($cNAD^+$) was synthesized from 5 mM cNMN and 5 mM ATP in the presence of 10 mM $MgCl_2$. The reaction was catalyzed by 0.5 U of *Ck*NMN-AT, facilitating the adenylyl transfer reaction.

Synthesis of $cNADP^+$ from $cNAD^+$

The conversion of $cNAD^+$ to carba-nicotinamide adenine dinucleotide phosphate ($cNADP^+$) was performed enzymatically using 5 mM $cNAD^+$, 2.5 mM ATP, and 10 mM $MgCl_2$. The same ATP regeneration system described above was applied. Phosphorylation was catalyzed by 0.46 U of *Ec*NADK.

Post-Reaction Processing

All reactions were performed at a 10 mL scale in a 10 mM HEPES buffer, pH 7.5, and incubated at 37 °C with shaking at 650 rpm for up to 20 hours. After incubation, the

reaction mixtures were processed using 10 kDa molecular weight cut-off (MWCO) centrifugal filtration units (Amicon® Ultra, Merck Millipore) to remove the enzymes. The filtrates, containing the products cNMN, $cNAD^+$, and $cNADP^+$, were then collected for downstream analytical purification and quantification.

Analytical Work-Up:

a) Purification

cNMN:

For the purification of cNMN, the aqueous, product-containing solution was applied to a cation exchange resin (Dowex 50W X8, H+-form, elution with H2O) for purification. The solvent was removed under reduced pressure and the residue dissolved in a small volume of H_2O. The pure product was obtained as yellowish foam, after freeze-drying. Product containing fractions were identified by TLC using a solvent mixture of propionic acid, water and 32% ammonia in ratio of 6.6/3.3/0.25 (R_f 0.45).

^1H NMR (400 MHz, D_2O) δ 9.41 (d, J = 1.8 Hz, 1H), 9.20 (dd, J = 6.4, 1.5 Hz, 1H), 8.96 (dd, J = 8.3, 1.6 Hz, 1H), 8.27 (ddd, J = 7.8, 5.9, 1.6 Hz, 1H), 5.12 (q, J = 9.4 Hz, 1H), 4.52 (ddd, J = 9.6, 5.6, 1.6 Hz, 1H), 4.21 (d, J = 5.6 Hz, 1H), 4.14 – 4.06 (m, 1H), 4.04 – 3.93 (m, 1H), 2.83 – 2.70 (m, 1H), 2.54 – 2.45 (m, 1H), 2.22 – 2.09 (m, 1H).

CarbaNAD$^+$:

For the purification of carbaNAD$^+$, the filtrate was applied to an anion-exchange resin (Dowex 1x8, formate-form; elution of product with 0.03 M formic acid) for purification. The product-containing fractions were combined and the solvent removed by freeze-drying. The product was obtained as white amorphous powder. Product containing fractions were

identified by TLC using a solvent mixture of propionic acid, water and 32% ammonia in ratio of 6.6/3.3/0.25 (R_f 0.21).

^{1}H NMR (400 MHz, D_2O) δ 9.42 (d, *J* = 1.5 Hz, 1H), 9.20 (dt, *J* = 6.4, 1.4 Hz, 1H), 8.93 (dt, *J* = 8.2, 1.7 Hz, 1H), 8.63 (s, 1H), 8.44 (s, 1H), 8.25 (dd, *J* = 8.1, 6.1 Hz, 1H), 6.16 (d, *J* = 5.4 Hz, 1H), 5.19 – 5.04 (m, 1H), 4.75 (t, *J* = 5.1 Hz, 1H), 4.59 – 4.49 (m, 2H), 4.46 – 4.37 (m, 1H), 4.34 – 4.23 (m, 3H), 4.22 – 4.16 (m, 1H), 4.13 – 4.06 (m, 1H), 2.89 – 2.66 (m, 1H), 2.55 – 2.42 (m, 1H), 2.29 – 2.17 (m, 1H).

CarbaNADP$^+$:

For the purification of carbaNADP$^+$, the filtrate was applied to an anion-exchange resin (Dowex 1x8, formate-form; elution of product with 0.25 M LiCl) for purification. Before application of the LiCl solution, the column was properly flushed with 0.6 M formic acid to remove side-products and unreacted carbaNAD$^+$. The product-containing fractions were combined and the solvent removed by freeze-drying. To remove the residual LiCl, the fractions were suspended in EtOH. The samples were centrifuged and the supernatant discarded. The solid material was dissolved in a minimum amount of water and precipitated in ice-cold EtOH. The samples were centrifuged again and the supernatant removed. The solid material was dissolved in water, frozen and freeze-dried. The product was obtained as white amorphous powder. Product containing fractions were identified by TLC using a solvent mixture of propionic acid, water and 32% ammonia in ratio of 6.6/3.3/0.25 (R_f 0.18).

^{1}H NMR (400 MHz, D_2O) δ 9.30 (s, 1H), 9.08 (d, *J* = 6.3 Hz, 1H), 8.85 – 8.78 (m, 1H), 8.19 (s, 1H), 8.15 (dd, *J* = 8.1, 6.2 Hz, 1H), 6.14 (d, *J* = 5.0 Hz, 1H), 5.07 – 4.95 (m, 2H), 4.64 – 4.57 (m, 1H), 4.51 – 4.42 (m, 1H), 4.41 – 4.36 (m, 1H), 4.35 – 4.27 (m, 1H), 4.25 – 4.16 (m, 2H), 4.16 – 4.10 (m, 1H), 4.08 – 4.01 (m, 1H), 2.65 – 2.53 (m, 1H), 2.43 – 2.38 (m, 1H), 2.18 – 2.05 (m, 1H).

b) LC-ESI-MS of Purified Products

The HPLC system (Ultimate 3000RS, Dionex) was composed of a degasser (SRD 3400), a pump module (HPG 3400RS), an autosampler (WPS 3000TRS), a column compartment (TCC 3000RS), a diode array detector (DAD 3000RS) and an ESI-ion-trap unit (HCT, Bruker). Data was collected and analyzed with Bruker Hystar, and Dionex Chromelion software. The column (Triat Hilic-Diol, 100 mm length, 2 mm i.d.; 1.9 µm particle size; YMC) was tempered to 7 °C. Mobile phase A consisted of 5 mM ammonium formate buffer (pH 4.5) and mobile phase B consisted of acetonitrile containing 0.1% v/v formic acid. The chromatographic flow rate was set to 0.4 mL/min. The gradient was programmed as following: start of mobile phase A at 15% for 2 min, with increase to 21% over 2 min, hold for 3 min, with following increase to 35% over 2 min. The gradient was hold for 3 min and returned to starting conditions. The starting conditions of mobile phase A at 15% was hold for 5 min. Before entering ESI-MS the flow was splitted 1:20 (Accurate-Post-Column-Splitter, Dionex). Temperature of the autosampler was set to 10 °C and an injection volume of 10 µL was used.

The ion-trap was operated in the ultra-scan mode (26,000 *m*/*z*/s) from 50 to 1500 *m*/*z*. The ICC target was set to 200,000 with a maximum accumulation time of 50 ms and four averages. The ion source parameters were set as follows: capillary voltage 4 kV, dry

temperature 325 °C, nebulizer pressure 40 psi and dry gas flow 6 L/min. Auto-MS-mode with the smart target mass of 600 *m*/*z* and a MS/MS fragmentation amplitude of 0.5 V was used. The quantification was performed by using the extracted ion chromatograms (EIC) of the *m*/*z* value corresponding to the protonated molecules.

c) NMR Spectroscopy

NMR spectra were measured on a JNM-ECA 400 MHz spectrometer from JOEL at 25 °C with the application of standard pulse programs. Chemical shifts (δ) are reported in ppm with reference to tetramethylsilane or the respective deuterated solvent. Coupling constants *J* are reported in Hertz (Hz). For determining CH_2-signals, the DEPT135°-technique was used.

1.5 Thermostability anaylsis : NAD^+ and $cNAD^+$

The thermal stability of NAD^+ and $cNAD^+$ was evaluated by incubation at elevated temperature followed by quantitative HPLC analysis. Stock solutions of each cofactor (10 mM) were prepared in 100 mM HEPES buffer (pH 7.5). Aliquots (1.0 mL) were transferred into 1.5 mL Eppendorf vials and incubated in an Eppendorf ThermoMixer C at 50 °C and 600 rpm in a horizontal position to ensure uniform heating and mixing. At defined time intervals (every 24 h up to 96 h), 50 µL aliquots were withdrawn and diluted with deionized water to 500 µL. For HPLC analysis, 100 µL of this diluted sample was mixed with 360 µL acetonitrile (100%) to obtain the final analytical solution.

Chromatographic separations were performed on a YMC Triart HILIC-Diol column (100 × 2.0 mm, 1.9 µm) using a flow rate of 0.4 mL min^{-1} and UV detection at 260 nm. The column temperature was maintained at 7 °C, and the autosampler temperature was set to 20 °C.

The injection volume was 10 µL. The mobile phase consisted of (A) 0.1% formic acid in water (pH 4.5) and (B) acetonitrile with 0.1% formic acid. Separation was achieved using a segmented linear gradient: 85% B held for 2 min, decreased to 79% B over 2 min, held for 1 min, further decreased to 70% B over 3 min and held for 2 min, followed by a return to 85% B and re-equilibration for 5 min. Retention times were 9.7 min for NAD^+ and 10.2 min for $cNAD^+$. Peak areas were integrated and normalized to the initial concentration (C_0).

Apparent first-order decay rate constants (ki) were determined from the slope of a linear fit of $\ln(C/C_0)$ versus incubation time according to $C=C_o.e^{-ki.t}$. Where Co is initial concentration of the cofactor, C: concentration at time t, t: incubation time, ki: apparent first-order decay rate constant.

1.6 Oxidoreductase Activity Assays with NAD(H) and cNAD(H)

For each enzyme, reactions were performed at a representative substrate concentration, and relative reaction rates were determined by direct comparison of cofactor turnover. Cofactor-dependent activity was monitored photometrically by following the formation or consumption of the reduced cofactors. Changes in absorbance were recorded at 340 nm for NADH and at 360 nm for cNADH. Reaction components were prepared in water or the appropriate buffer and combined in UV-transparent 96-well microtiter plates (UV STAR®, 96-well, half-area, Greiner Bio-One, 675801) according to the compositions listed in Table S1. Mixing was achieved by pipetting, and reactions were conducted in a total volume of 100 µL. Absorbance measurements were collected at 1 min intervals using either an Epoch 2 microplate reader (BioTek) or an Infinite M200 microplate reader (Tecan). Reduced cNADH was generated enzymatically prior to activity measurements. For this

purpose, 200 µL reaction mixtures containing 5 mM D-galacturonic acid, 5 mM $cNAD^+$, 0.24 g L^{-1} *Agrobacterium tumefaciens* uronate dehydrogenase (*At*UDH), and 50 mM Tris buffer (pH 8.0) were incubated for 3 h at 30 °C in 96-well plates. Following incubation, AtUDH was removed by centrifugal filtration (3000 x g, 5 min) using 10 kDa polyethersulfone spin filters (VWR, 516-0230). UV–vis spectra confirmed approximately 85 % conversion of $cNAD^+$ to cNADH. As a result, assays initiated with cNADH contained a small residual fraction of oxidized $cNAD^+$. All enzymatic assays were performed in triplicate. For reactions initiated with NADH or cNADH, enzyme-free negative controls were included to account for non-enzymatic absorbance changes. Minor background signals observed in these controls were subtracted from the corresponding enzymatic reaction traces to ensure that reported rates reflect enzyme-dependent activity. Some enzymes had been stored at −80 °C for extended periods and may have exhibited partial activity loss. However, as the objective of this study was to compare relative cofactor acceptance rather than determine absolute specific activities, all comparisons were performed using matched conditions within each enzyme–cofactor pair.

2. Supplementary Figures

Figure S1

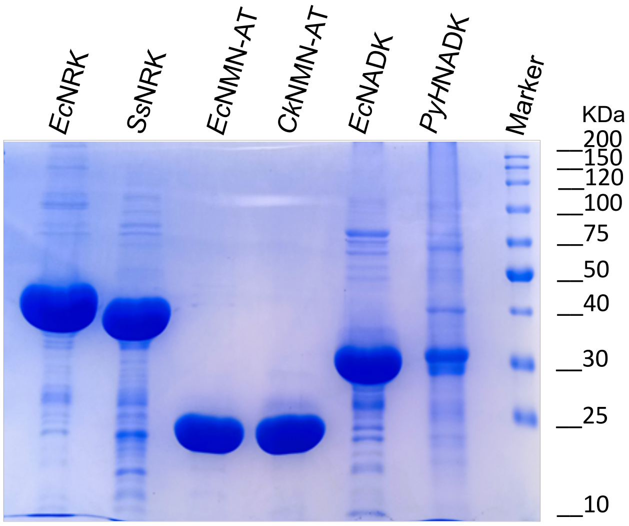

Figure S1: SDS–PAGE analysis of recombinant enzymes used in this study. SDS–PAGE analysis showing purified recombinant enzymes employed in the enzymatic synthesis of carba-nicotinamide cofactors. Lanes (from left to right) contain *E. coli* NRK (*Ec*NRK), *Streptococcus sanguinis* NRK (*Ss*NRK), *E. coli* NMNAT (*Ec*NMNAT), *Citrobacter koseri* NMNAT (*Ck*NMNAT), *E. coli* NADK (*Ec*NADK), and *Pyrococcus horikoshii* NADK (*Pyh*NADK). The rightmost lane contains the molecular-weight marker (Precision Plus Protein™ All Blue Prestained Protein Standard, Bio-Rad).

Figure S2 : Size-exclusion chromatography (SEC) analysis of recombinant enzymes.

(a)

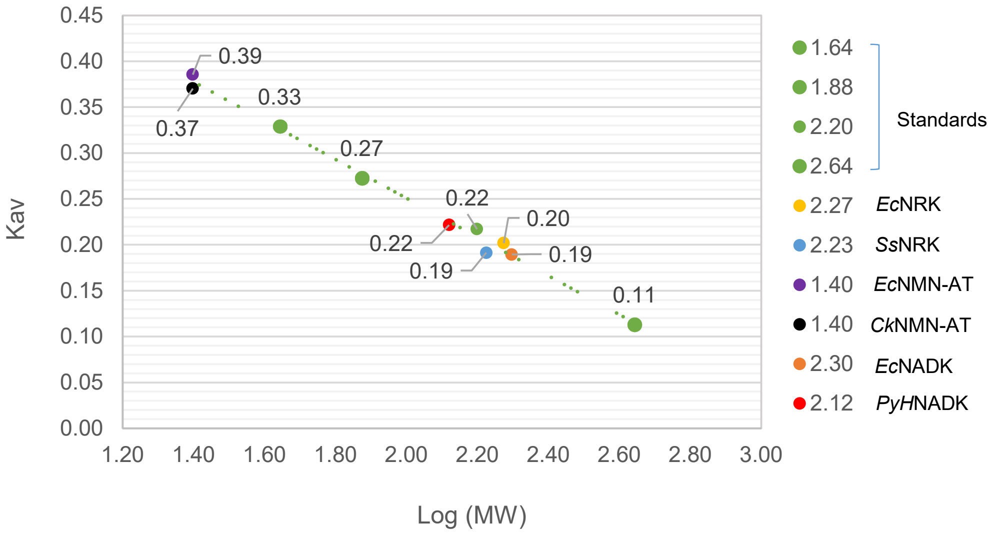

(b)

Enzyme	Ve	Kav	MW (monomer) KDa	Calculated (MW)	Theoretical (MW)	Quaternary structure
EcNRK	14.51	0.20	47	172	188	Tetramer
SsNRK	14.23	0.19	42	193	168	Tetramer
EcNMN-AT	19.34	0.39	25	23	25	Monomer
CkNMN-AT	18.94	0.37	25	28	25	Monomer
EcNADK	14.18	0.19	33	197	198	Hexamer
PyHNADK	15.03	0.22	33	139	132	Tetramer

Figure S2: Size-exclusion chromatography (SEC) calibration and analysis of recombinant enzymes. (a) Calibration curve generated by plotting the partition coefficient (K_{av}) against the logarithm of molecular weight (log MW) for standard proteins . The curve was used to estimate native molecular weights of the purified enzymes from their elution volumes (Ve). (b) Summary of SEC-derived parameters, including elution volume (Ve), calculated Kav, estimated molecular weight, and inferred quaternary structure for each enzyme. Both NRK variants eluted at tetramers, NMN-AT variants as monomers, and NADK enzymes eluted as hexamer and a tetramer.

Figure S3

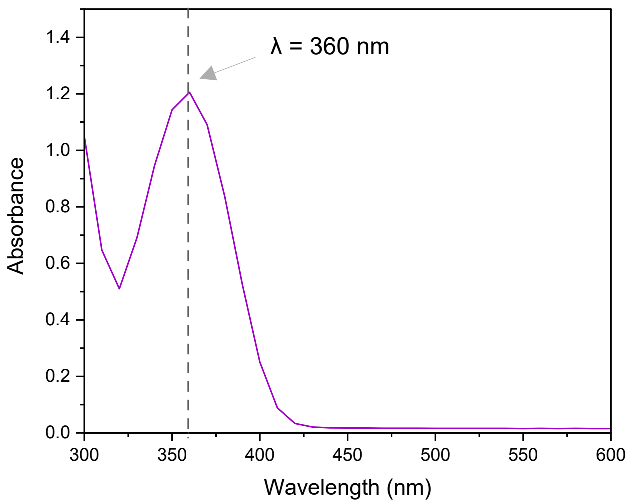

Figure S3: UV–Vis spectral scan of cNADH. The absorbance spectrum of enzymatically generated cNADH shows a maximum at 360 nm, which was used as the detection wavelength for spectroscopic assays in this study.

Figure S4

(a) **Overlay of Samples from Integration View**

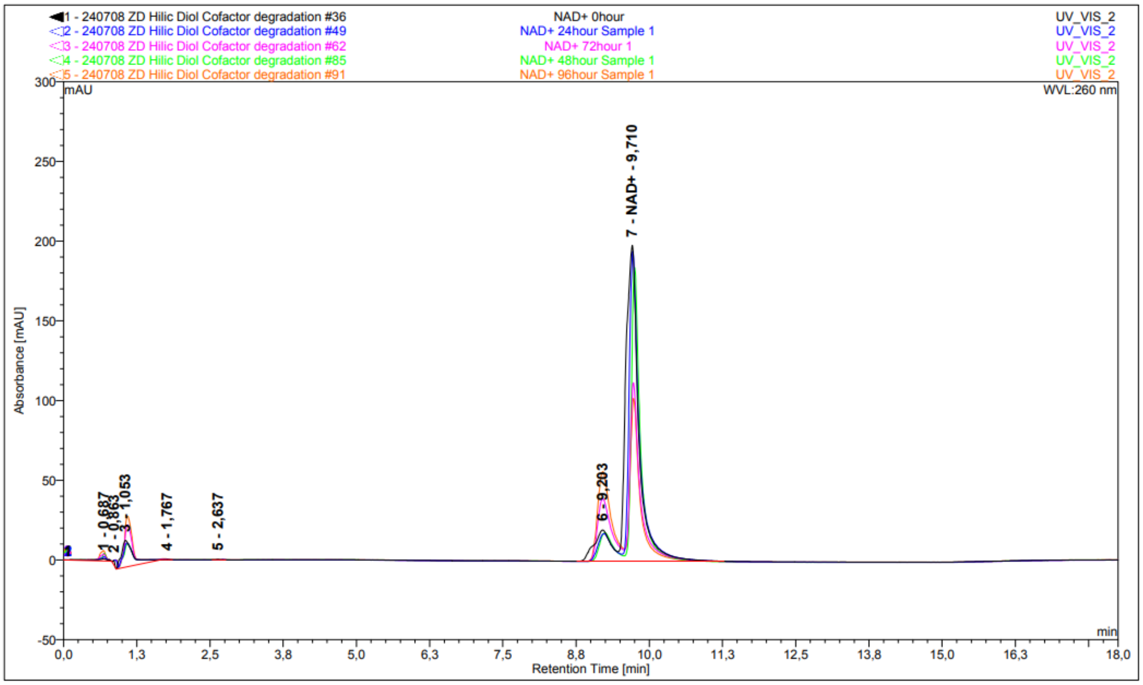

(b) **Overlay of Samples from Integration View**

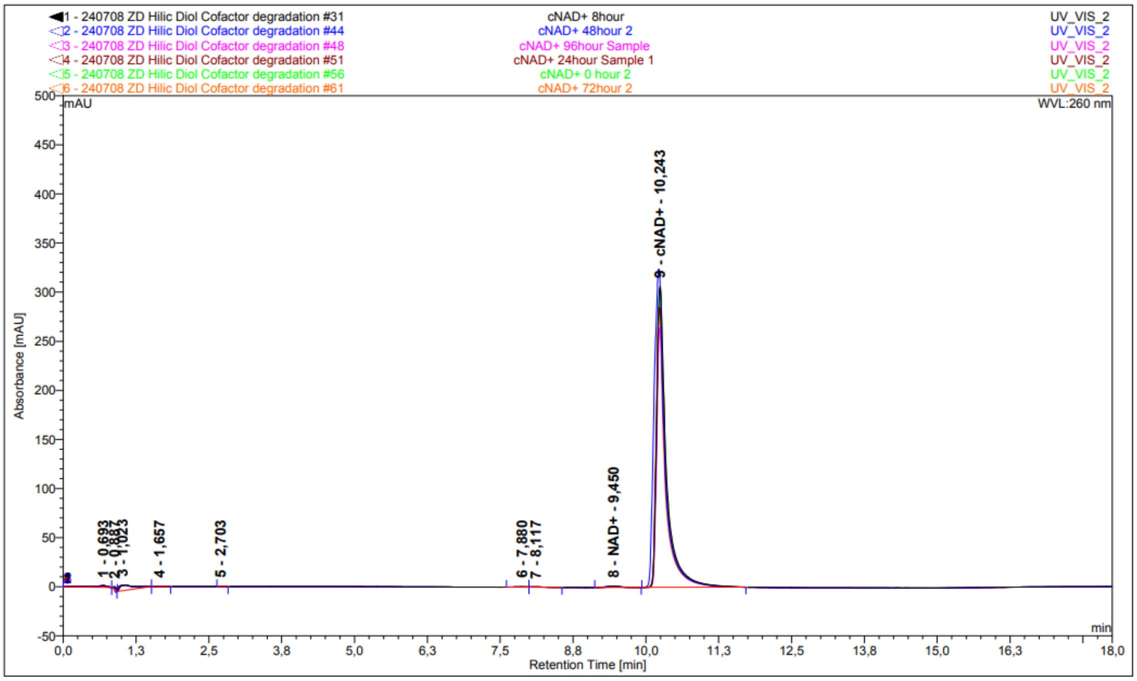

Figure S4: Hydrolysis profiles of NAD^+ and $cNAD^+$. Overlay of HPLC chromatograms collected at defined time points during incubation at 50 °C (0–96 h), showing the progressive degradation of (a) NAD^+ and (b) $cNAD^+$. NAD^+ exhibits the appearance of multiple degradation products over time, whereas $cNAD^+$ retains a single dominant peak throughout the experiment, indicating high thermal stability.

Figure S5

1) **CarbaNADP$^+$**: UV-chromatogram; m/z + fragmentation analysis

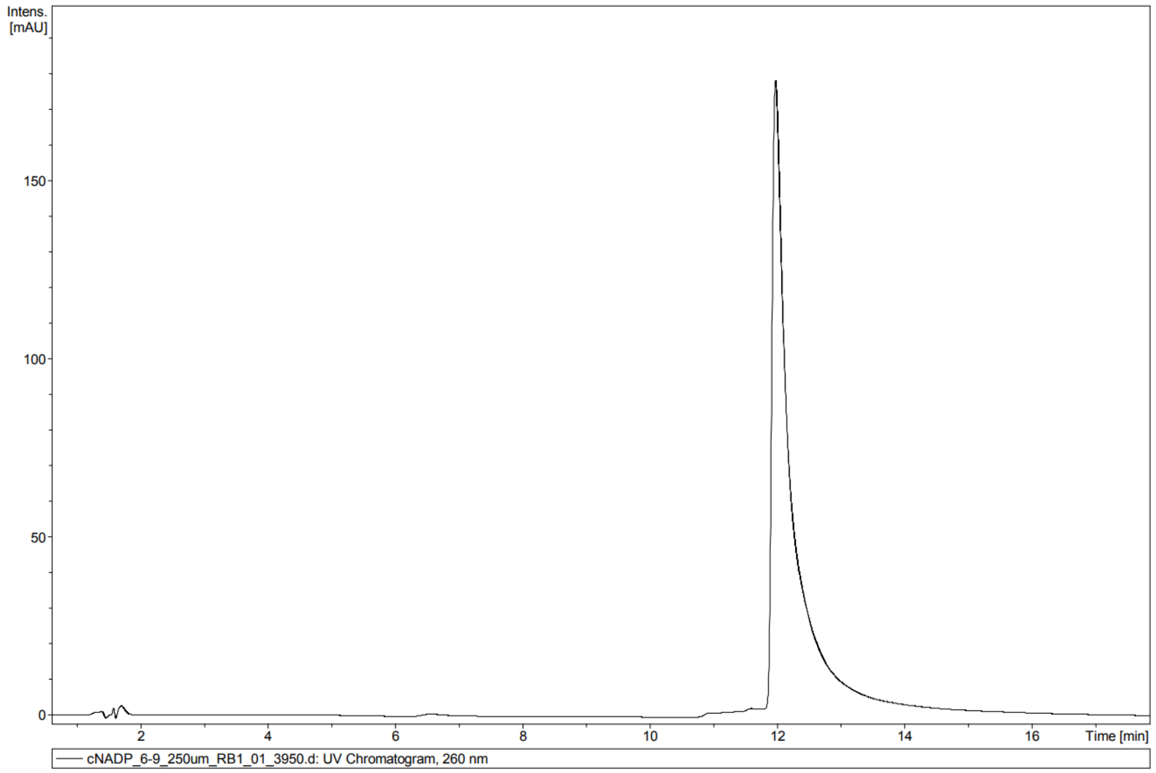

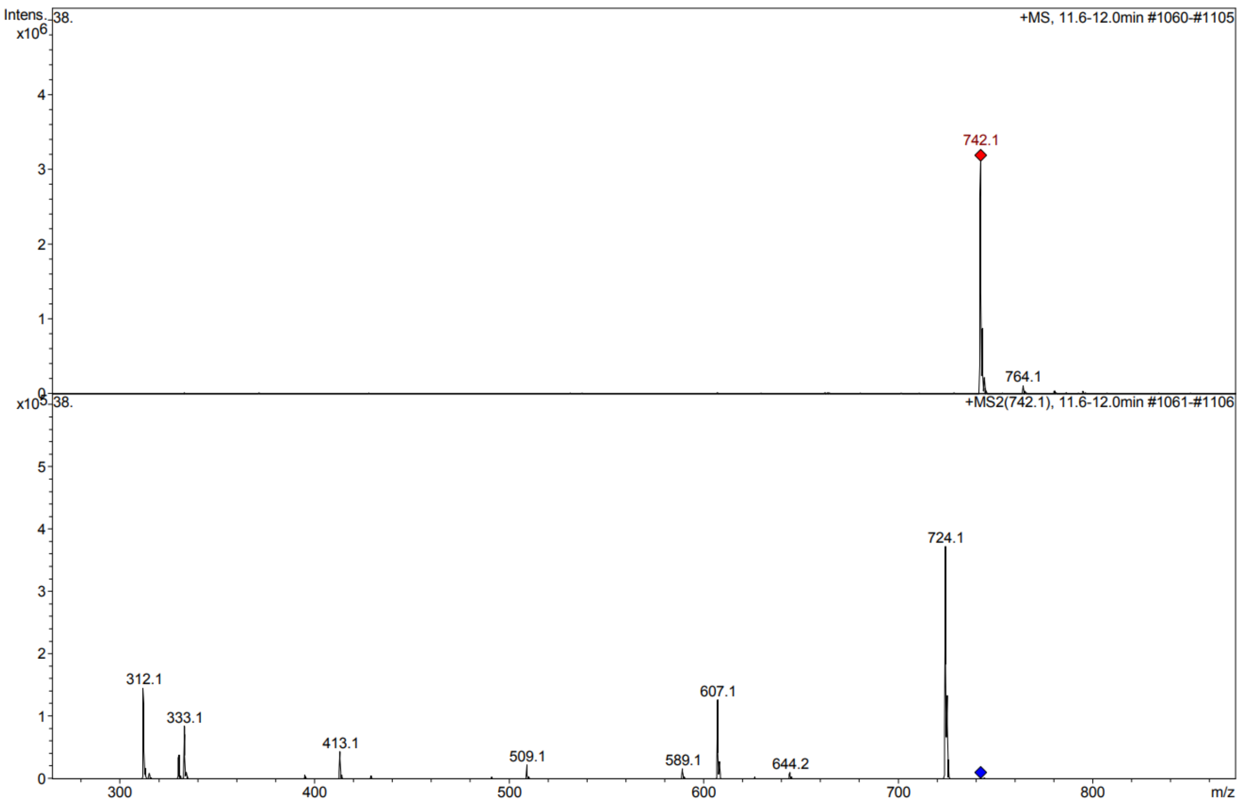

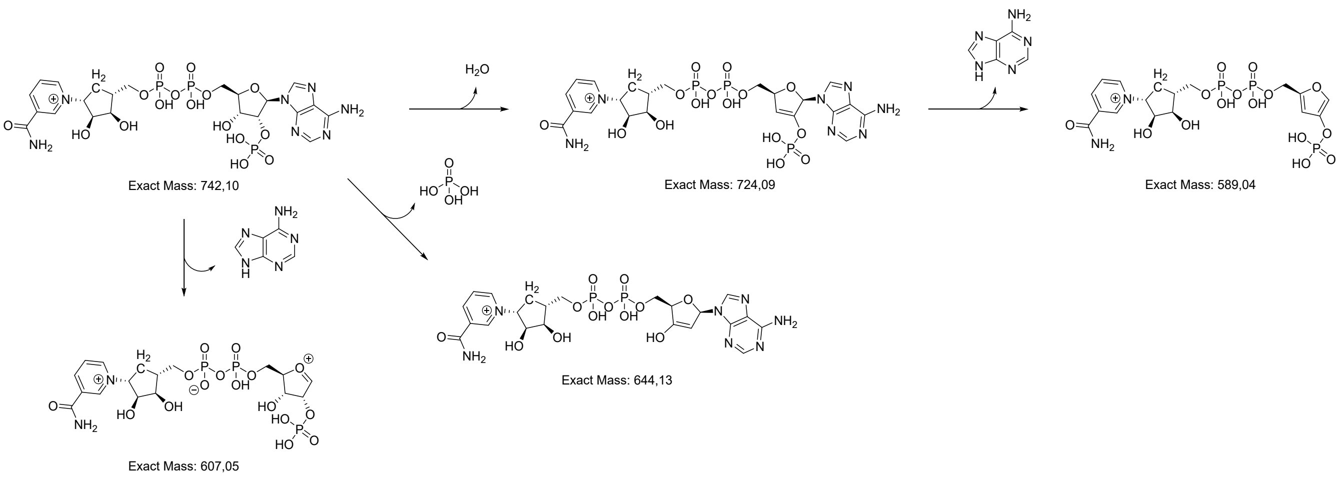

Scheme: cNADP+ m/z Fragmentation analysis

2) CarbaNAD^{+}: UV-chromatogram; m/z + fragmentation analysis

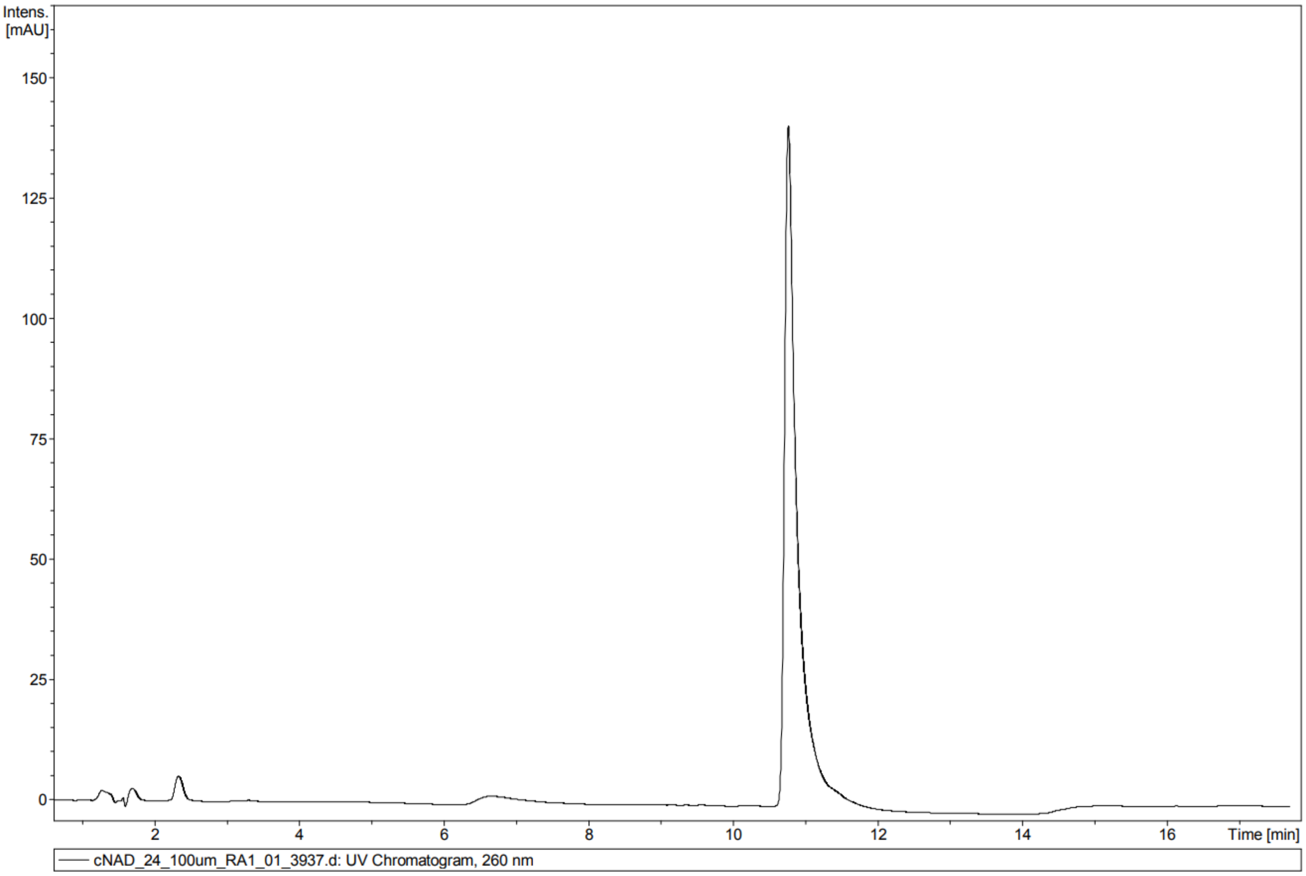

Intens.
[mAU]
150
125
100
75
50
25
0
2
4
6
8
10
12
14
16
Time [min]
cNAD_24_100um_RA1_01_3937.d: UV Chromatogram, 260 nm

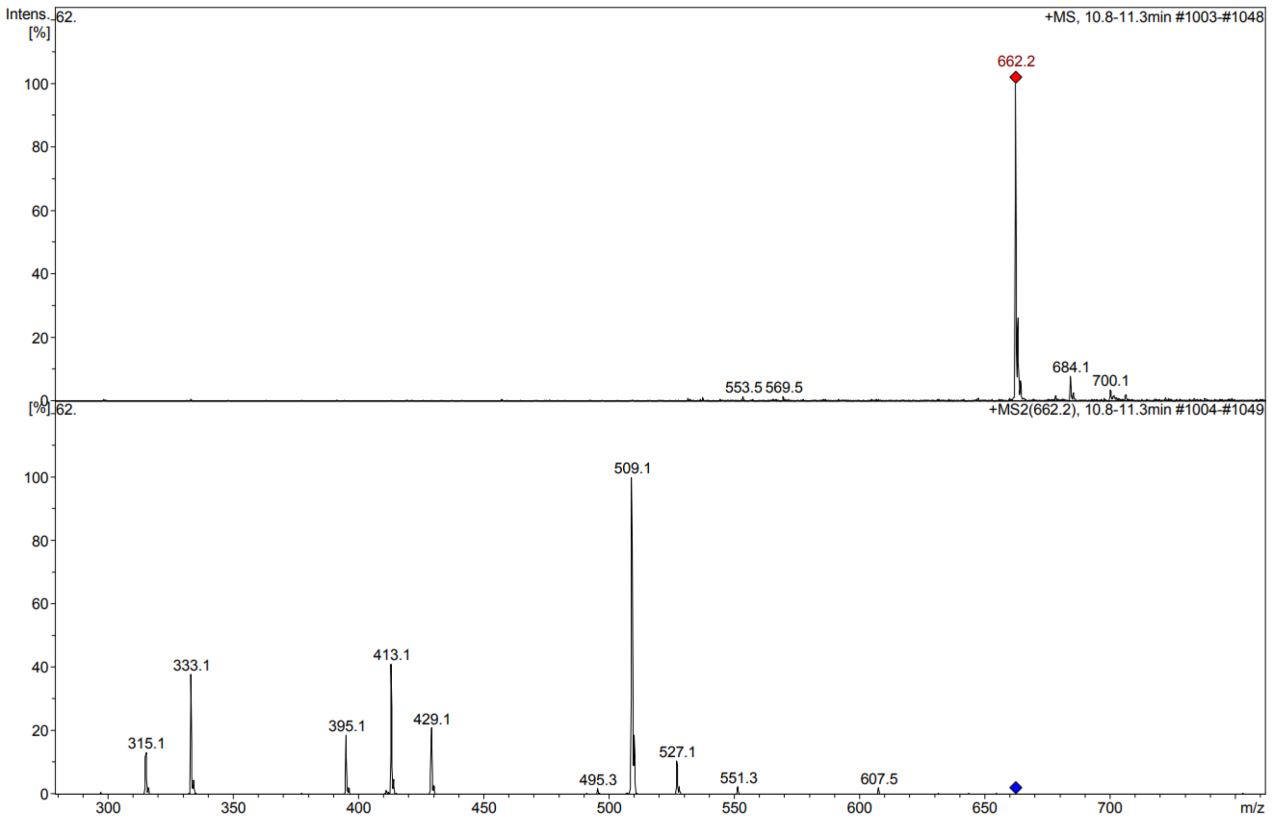

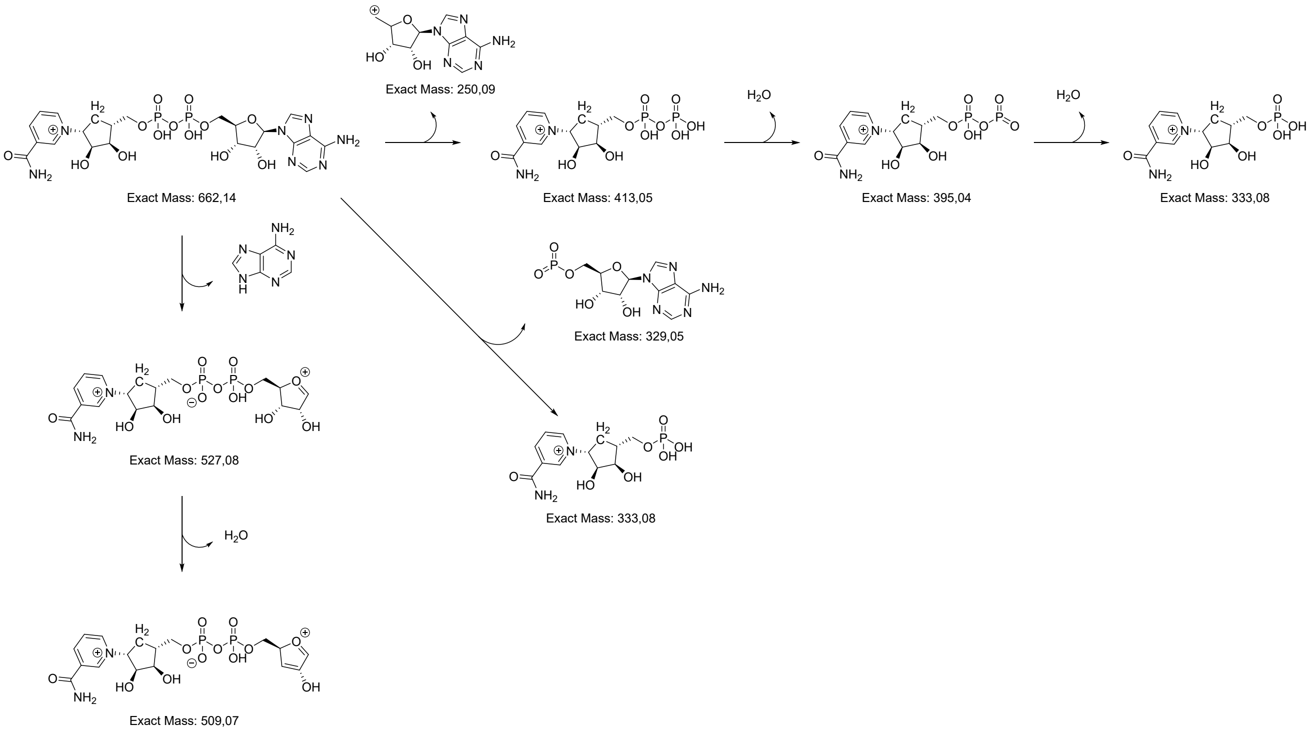

Scheme: cNAD+ m/z Fragmentation analysis

3) cNMN: UV-chromatogram; m/z + fragmentation analysis

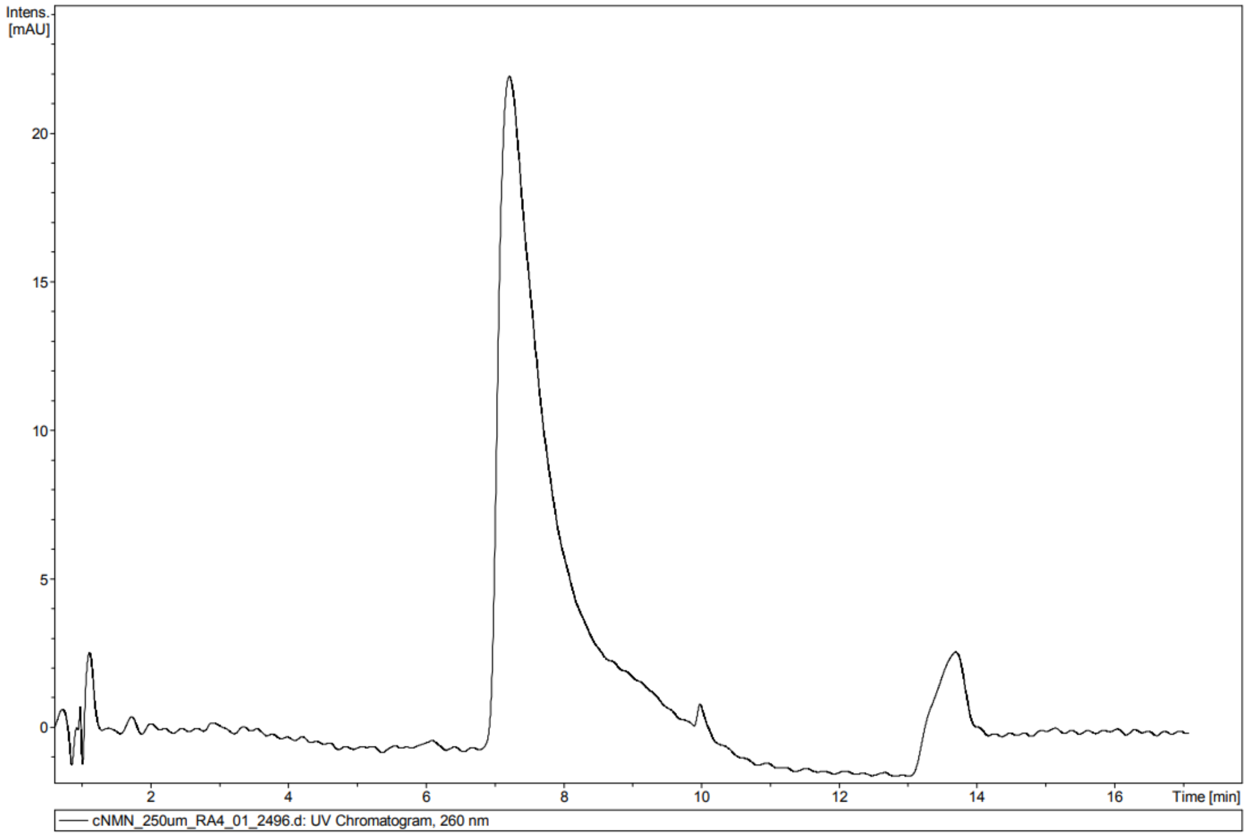

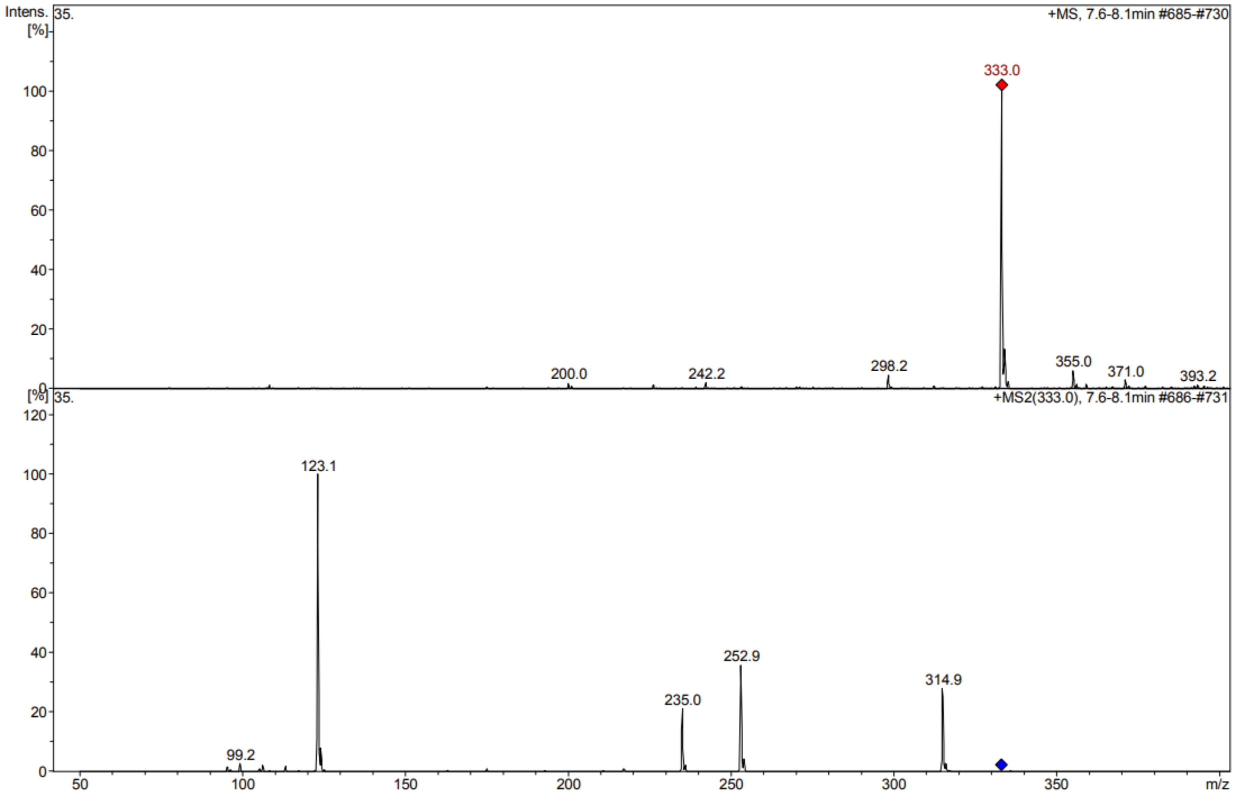

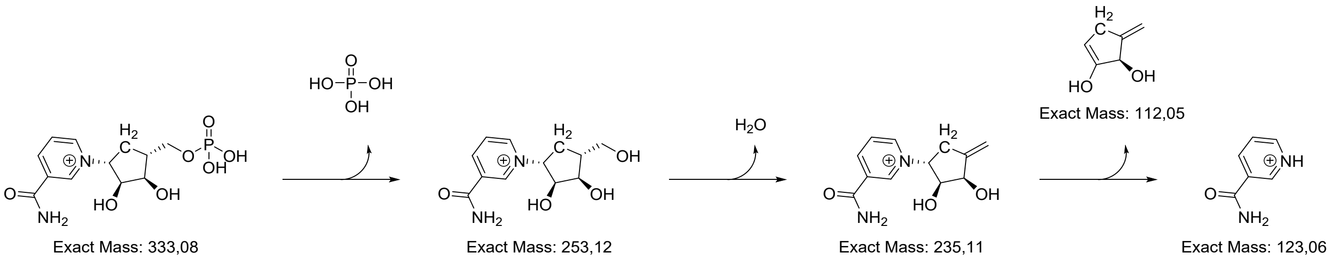

Scheme: cNMN m/z Fragmentation analysis

3. NMR spectra

Figure S6

CarbaNADP$^+$: ^{1}H-spectrum (signals at 3.53 and 1.06 ppm belong to residual EtOH)

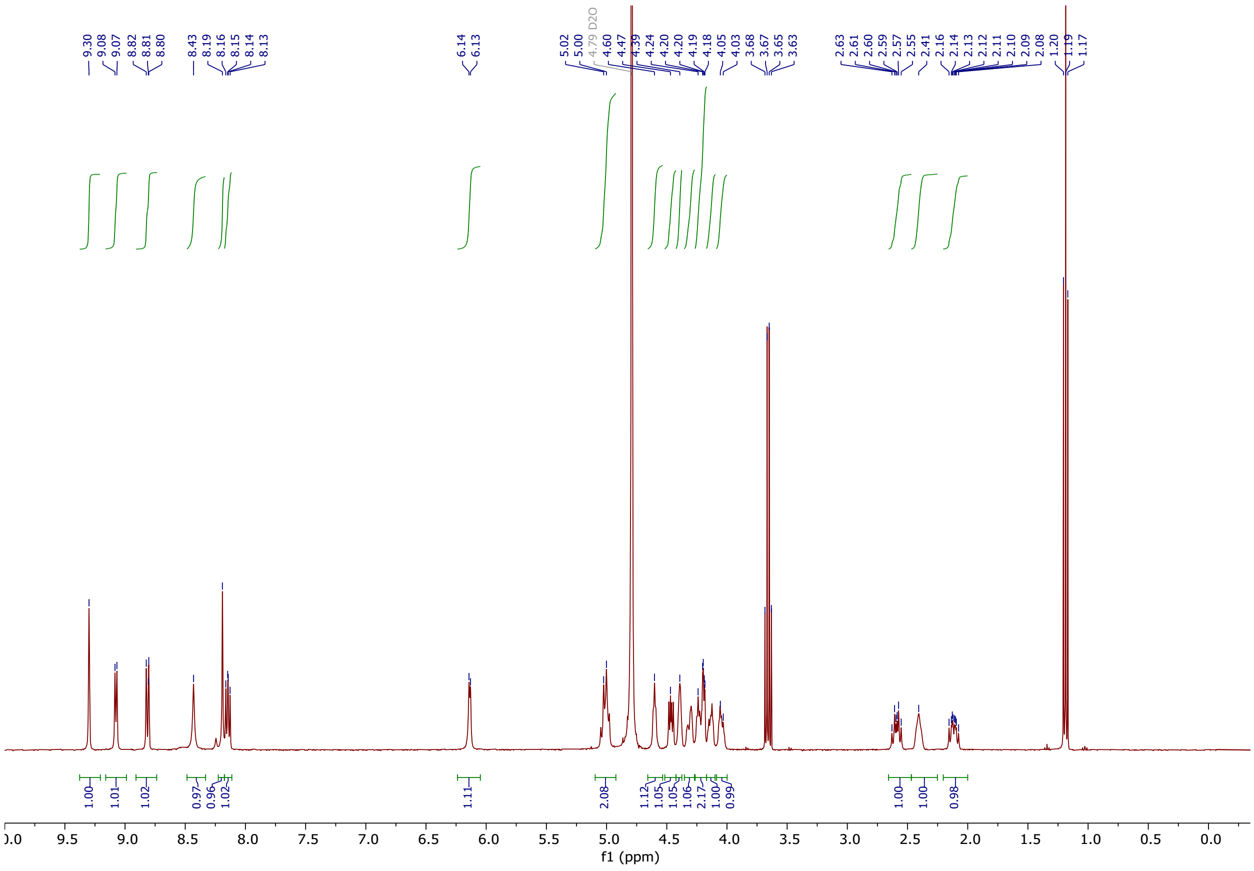

^{1}H-spectrum of carbaNADP+.

CarbaNAD+: ^{1}H-spectrum

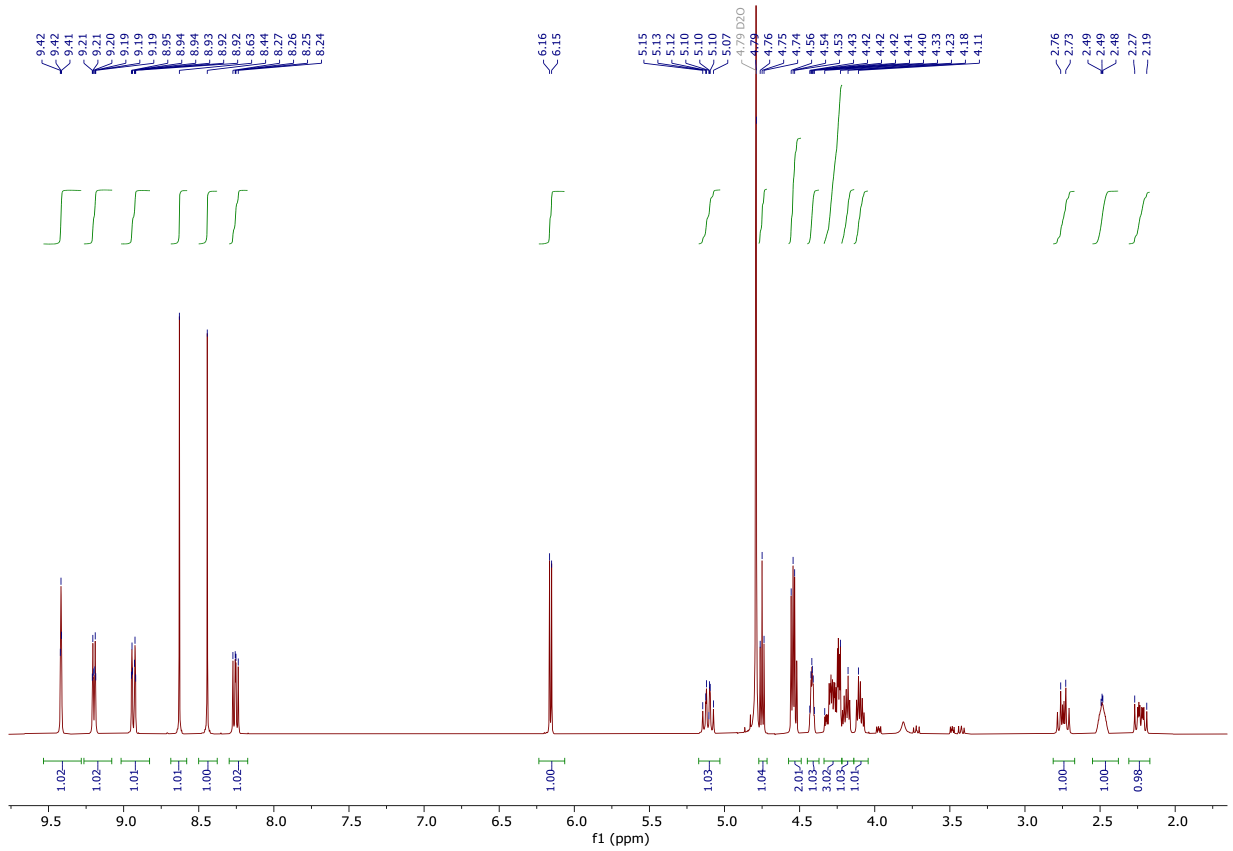

cNMN: ^{1}H-spectrum

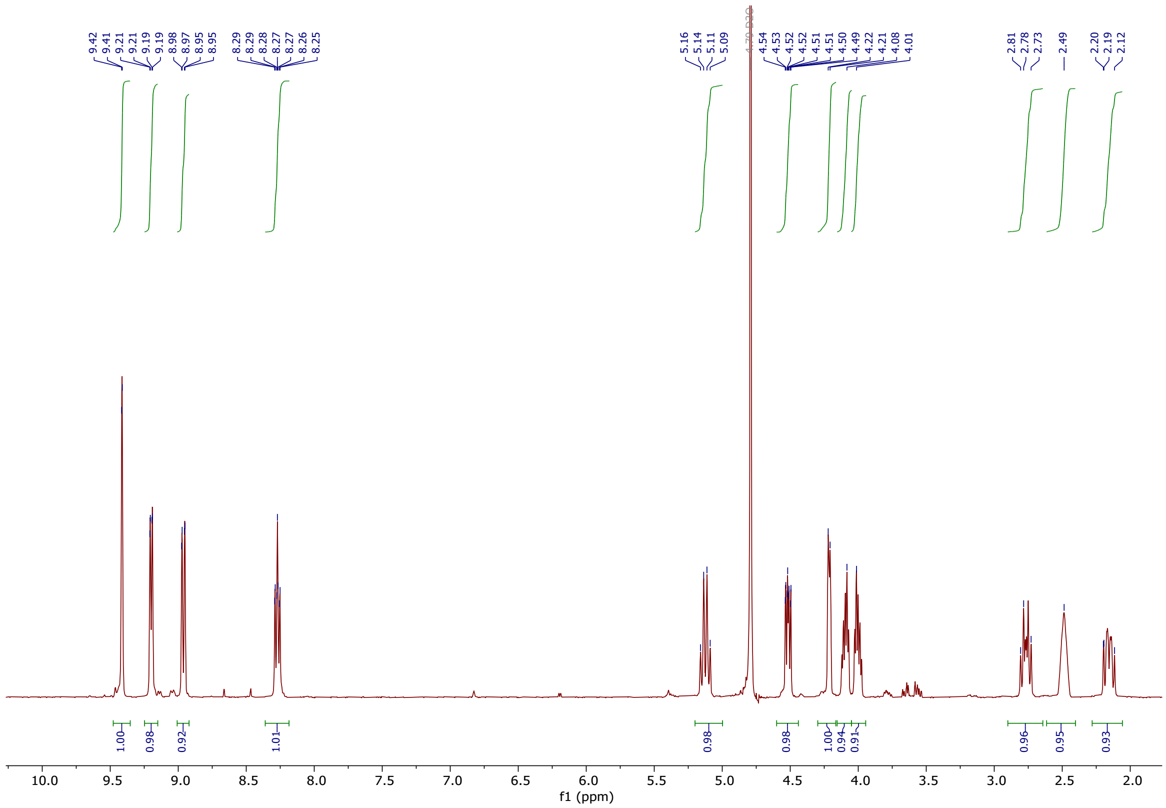
9.42
9.41
9.21
9.21
9.19
9.19
8.98
8.97
8.95
8.95
8.29
8.29
8.28
8.27
8.27
8.26
8.25
5.16
5.14
5.11
5.09
4.79 D2O
4.54
4.53
4.52
4.52
4.51
4.51
4.50
4.49
4.22
4.21
4.08
4.01
2.81
2.78
2.73
2.49
2.20
2.19
2.12
1.00
0.98
0.92
1.01
0.98
0.98
1.00
0.94
0.91
0.96
0.95
0.93
f1 (ppm)

Figure S7

NMR Spectra – Chemical synthesis of cNAD⁺, Intermediates

(1) (1R,4S,5R,6S)-5,6-Dihydroxy-2-azabicyclo[2.2.1]heptan-3-one

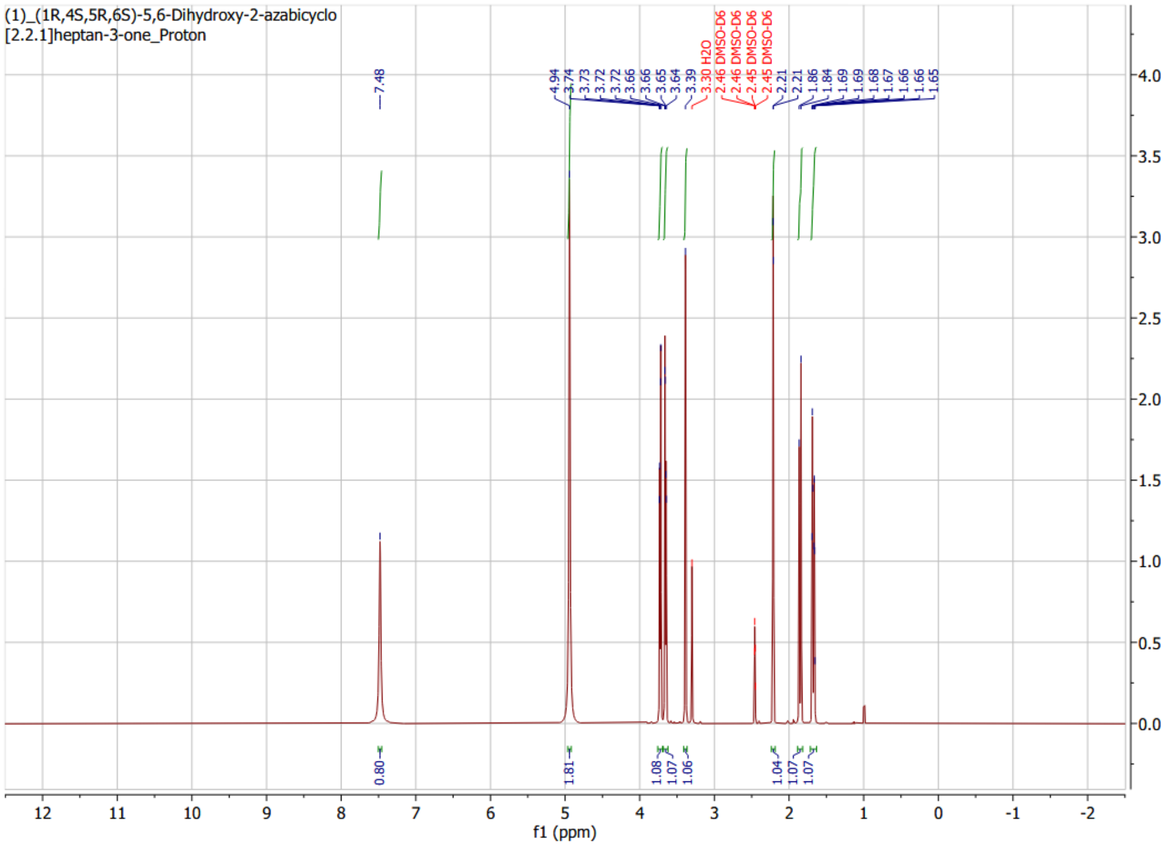

(2) (3aS,4R,7S,7aR)-2,2-dimethyltetrahydro-4,7-methano[1,3]dioxolo[4,5-c]pyridine-6(3aH)

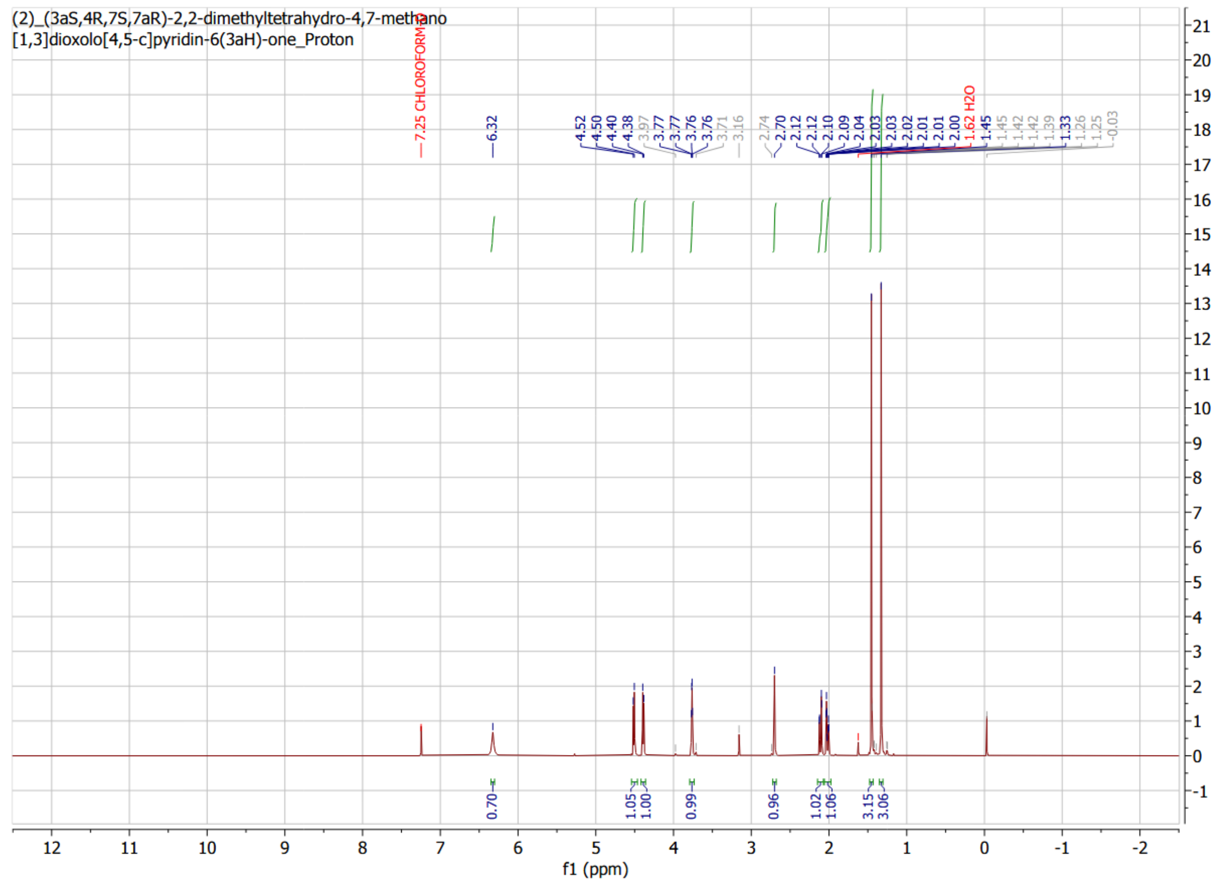

(3) *tert*-Butyl (3aS,4S,7S,7aR)-2,2-dimethyl-6-oxotetrahydro-4,7-methano[1,3]dioxolo[4,5-c]pyridine-5(4H)-carboxylate

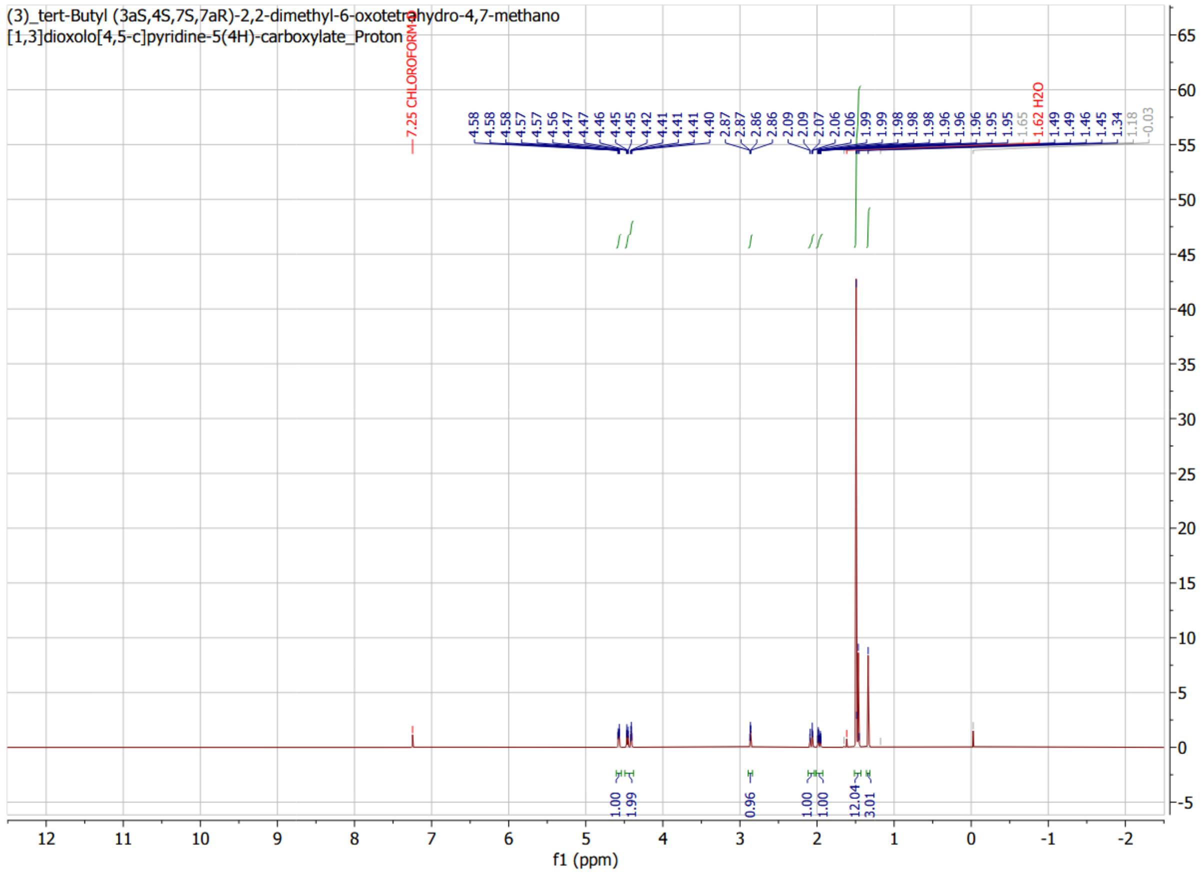

(4) *tert*-butyl ((3aS,4R,6R,6aR)-6-(hydroxymethyl)-2,2-dimethyltetrahydro-4H-cyclopenta[d]

[1,3]dioxol-4-yl)carbamate

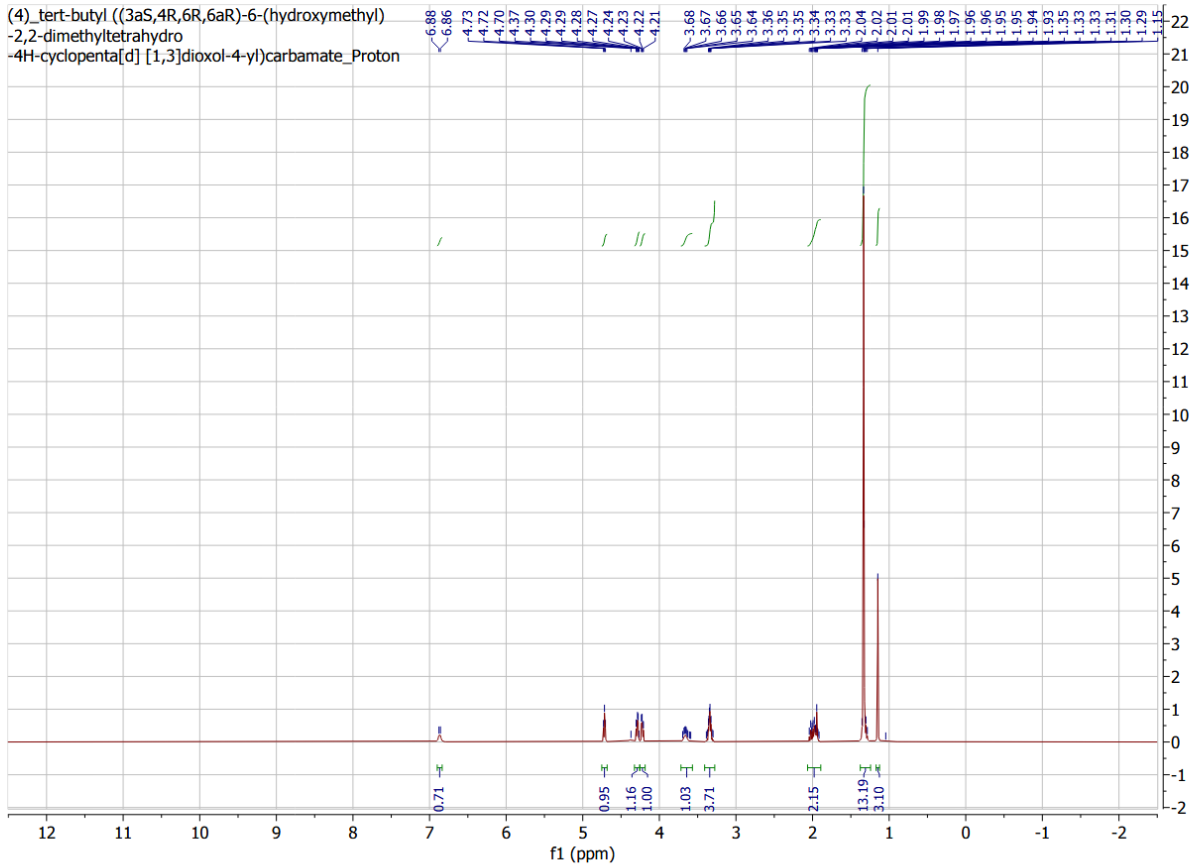

(5) (1R,2S,3R,5R)-3-amino-5-(hydroxymethyl)cyclopentane-1,2-diol

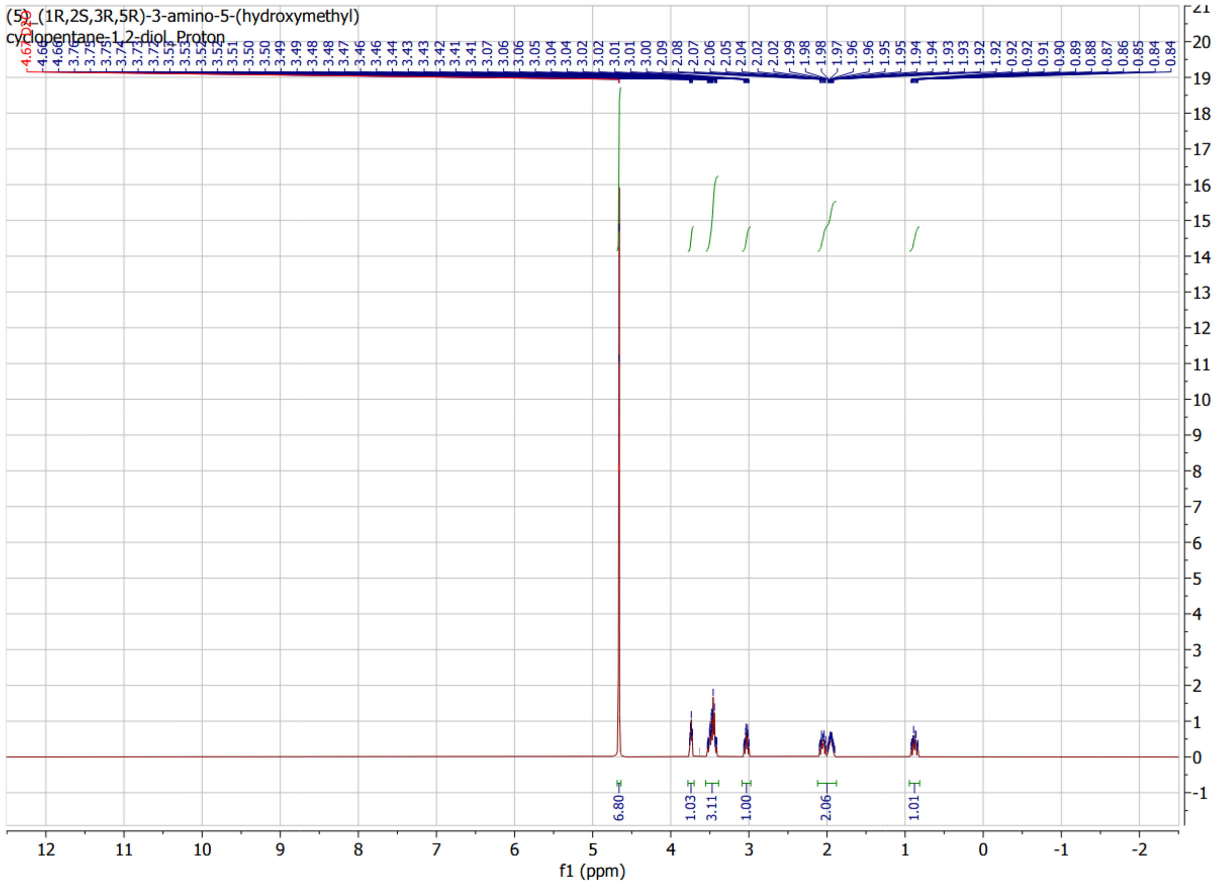

(6) 3-Carbamoyl-1-((1R,2S,3R,4R)-2,3-dihydroxy-4-(hydroxymethyl)cyclopentyl)pyridin-1-ium chloride

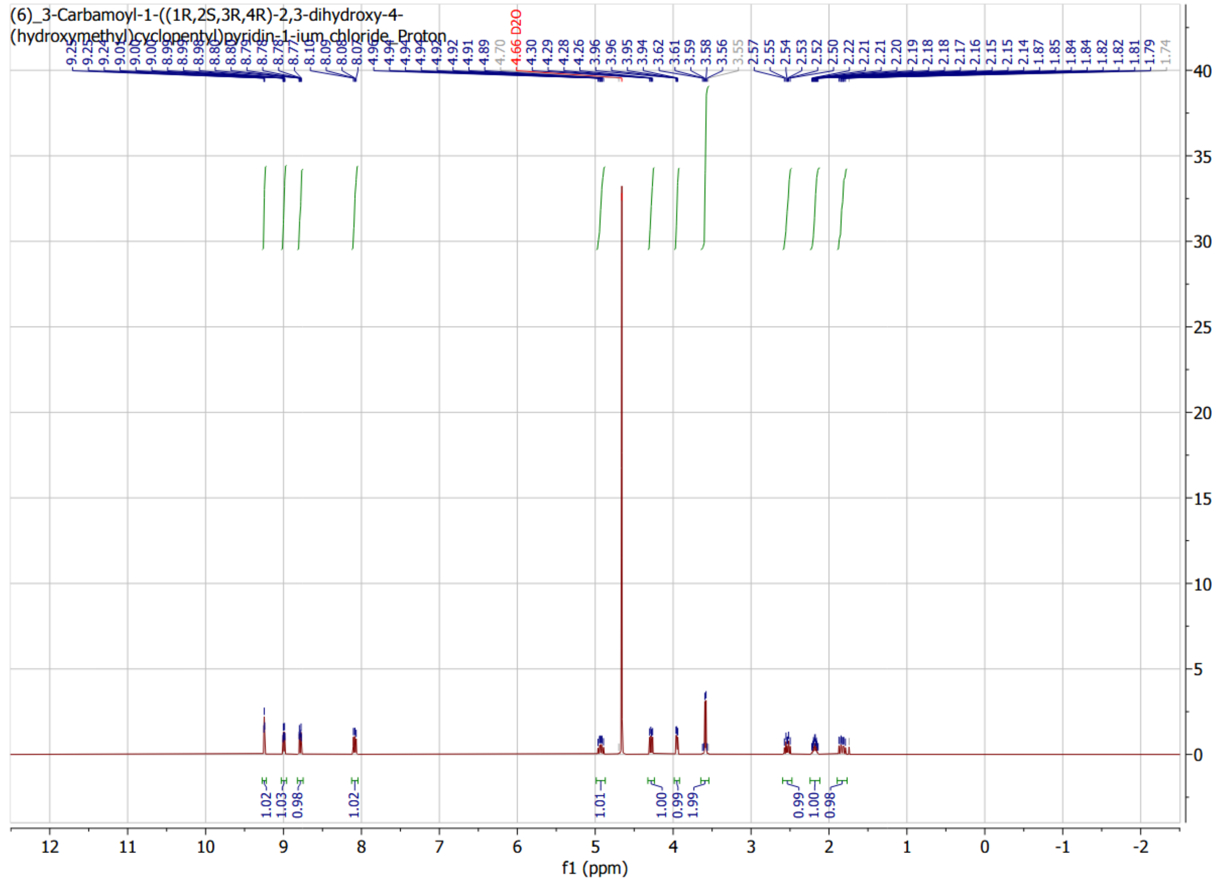

(7) 3-Carbamoyl-1-(2,4-dinitrophenyl)pyridine-1-ium chloride

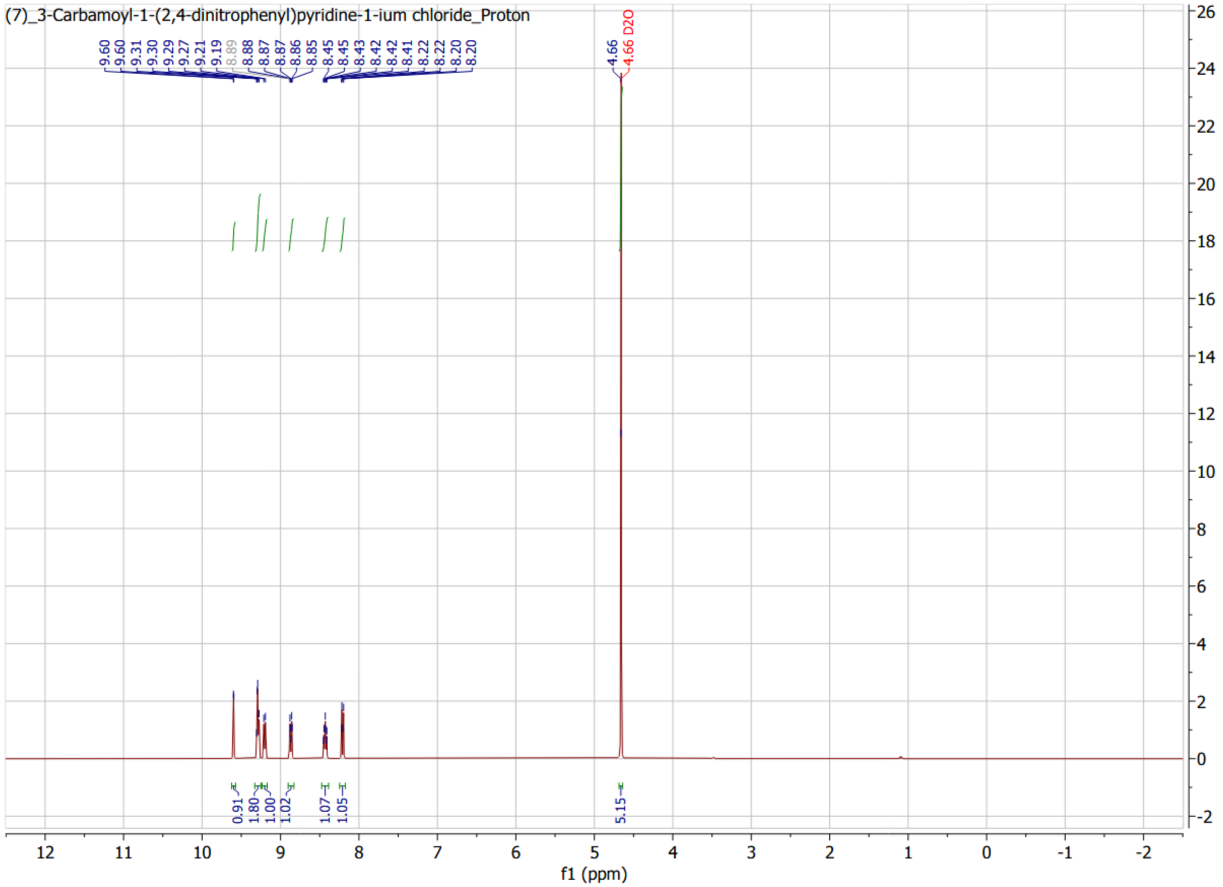

(8) ((1R,2R,3S,4R)-4-(3-carbamoylpyridin-1-ium-1-yl)-2,3-dihydroxycyclopentyl)methyl hydrogen phosphate

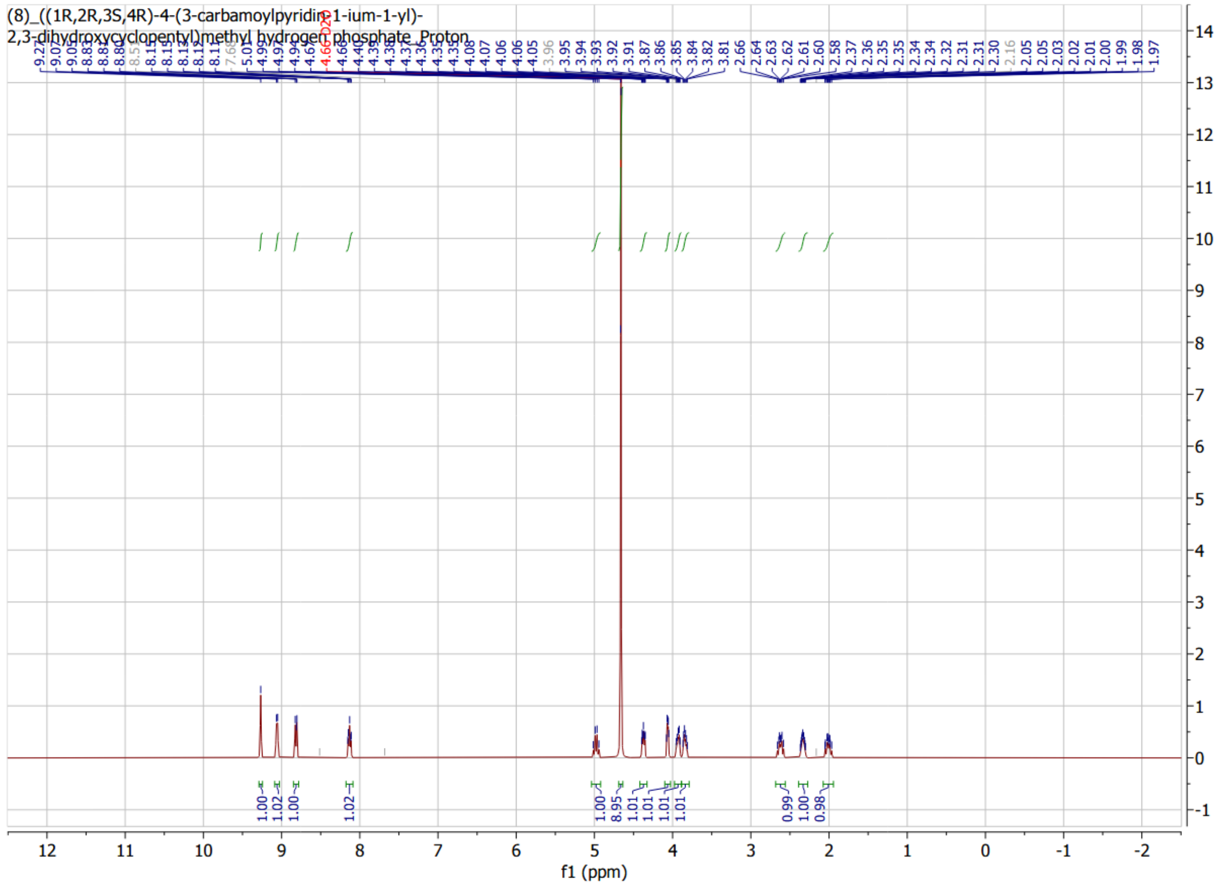

4. Supplementary Table

Table S1: List of oxidoreductases tested for cofactor acceptance, including specific activities measured with NAD(H), NADP(H), and cNAD(H), together with the corresponding substrates, reaction compositions, and assay conditions.

Entry	E. C. number	Enzyme	Enzyme name / UniprotKB or Accession No.	Organism	with NAD(H): [U x mg^{-1}]	with carba NAD(H): [U x mg^{-1}]	with NADP(H): [U x mg^{-1}]	NAD(H) / NAD(H)	carba NAD(H) / NAD(H)	NADP(H) / NAD(H)	Substrate	Reaction	Temperature [°C]	Buffer	pH	Substrate Conc. [mM]
1	1.1.1.1	Alcohol Dehydrogenase	*Pfu*ADH (N-His) / O 73949	*Pyrococcus furiosus*	0.0066	0.0150	0.0068	1.00	2.28	1.04	Benzaldehyde	Phenylmethanol + NAD(P)$^+$ <> Benzaldehyde + NAD(P)H	42	KPi	7.5	10
2	1.1.1.1	Alcohol Dehydrogenase	*Gst*ADH (N-His) / P 42328	*Geobacillus stearothermophilus*	2.5201	0.9353	0.0520	1.00	0.37	0.02	Benzaldehyde	Phenylmethanol + NAD(P)$^+$ <> Benzaldehyde + NAD(P)H	42	MES	6.5	10
3	1.1.1.1	Alcohol Dehydrogenase	*At*ADH	*Agrobacterium tumefaciens*	0.1105	9.8142	0.1087	1.00	88.78	0.98	FFCA	FFCA + (c)NADH <> 5HMF + (c)NAD	25	Tris-HCl	8	2
4	1.1.1.1	Alcohol Dehydrogenase	*Ssp*ADH / A0A075B5H4	*Sphingomonas spec.*	0.1082	0.0020	0.0105	1.00	0.02	0.10	D-Gluconate	D-Gluconate + (c)NAD <> L-Guluronate + (c)NADH	30	Tris-HCl	8	5
5	1.1.1.27	Lactate Dehydrogenase	LDH (Carl Roth)	Porcine	4.6231	54.4237	4.1917	1.00	11.77	0.91	Pyruvate	(s)-lactate + NAD$^+$ <> pyruvate + NADH	30	Tris-HCl	8	5
6	1.1.1.47	Glucose-1-Dehydrogenase	*Ss*Glucose DH (C-His) / O93715	*Sulfolobus solfataricus*	1.1051	0.1470	0.3261	1.00	0.13	0.30	**D-Glucose**, D-Xylose, D-Galactose, L-Arabinose	D-Glucose + NAD(P)$^+$ <> Glucono-d-lacton + NAD(P)H	42	Tris-HCl	8	5
7	1.1.1.49	Glucose-6-Dehydrogenase	*Lm*Glucose-6-Phosphate DH /	*Leuconostoc mesenteroides*	393.4	101.6	226.8	1.00	0.26	0.58	Glucose-6-Phosphate	Glucose-6-Phosphate + NADP$^+$ <> 6-phospho-D-glucono-1,5-lactone + NADPH	30	Tris-HCl	8	5

			P11411													
8	1.1.1.203	Uronate Dehydrogenase	AtUronate DH / Q7CRQ0	*Agrobacterium tumefaciens*	87.3	14.4	4.7	1.00	0.16	0.05	D-Galacturonate / Glucoronic acid	Beta-D-galacturonate + NAD^+ <> D-galactaro-1,5-lactone + NADH	30	Tris-HCl	8	5
9	1.1.1.203	Uronate Dehydrogenase	*Sv*Uronate DH / ZP_07302919.1	*Streptomyces viridochromogenes*	24.8	3.3	3.5	1.00	0.13	0.14	D-Galacturonate / Glucoronic acid	Beta-D-galacturonate + NAD^+ <> D-galactaro-1,5-lactone + NADH	30	Tris-HCl	8	5
10	1.2.1.9	Glyceraldehyde Dehydrogenase	*Tk*Glyceraldehyd-3-Phosphat DH	*Thermococcus kodakarensis*	0.5159	0.0066	0.7657	1.00	0.01	1.48	Glyceraldehyde-3-Phosphate	Glyceraldehyd-3-Phosphate + (c)NAD <> 3-Phosphoglycerat + (c)NADH	30	HEPES	7.5	1
11	1.4.1.21	Aspartate Dehydrogenase	*Vp*AspartatDH	*Variovorax paradoxus*	132.1	148.3	56.8	1.00	1.12	0.43	Oxalacetate	Oxalacetate + (c)NADH --> Aspartat + (c)NAD+	30	HEPES	7.5	5
12	1.4.1.9	Leucine Dehydrogenase	*Bs*LeuDH	*Bacillus subtilis*	91.1	114.9	1.8	1.00	1.26	0.02	2-Ketoisovalerate	Ketoisovalerate + (NH4)2SO4 +(c)NADH --> Valin + (c)NAD+	30	HEPES	7.5	1
13	1.1.1.49	Glucose-6-Dehydrogenase	*Ec*Glucose-6-Phosphate DH / P11411	*Escherichia coli*	20.056	0.027	53.321	1.00	0.00	2.66	Glucose-6-Phosphate	Glucose-6-Phosphate + $NADP^+$ <> 6-phospho-D-glucono-1,5-lactone + NADPH	30	Tris-HCl	8	5
14	1.1.1.86	Ketol-acid Reductoisomerase	*Gs*Ilvc (KARI)	*Geobacillus stearothermophilus*	0.0139	0.0037	0.0535	1.00	0.26	3.85	Acetolactate	(s)-2-Acetolactate + (c)NADH <> 2,3-Dihydroxyisovalerate + (c)NAD+	30	HEPES	7.5	1
15	1.4.1.3	Glutamate Dehydrogenase	L-Glutamic DH / P00366	Bovine liver	0.2714	0.0273	0.2381	1.00	0.10	0.88	L-Glutamate	L-glutamate + $NAD(P)^+$ <> a-ketoglutarate + NAD(PH)H + NH_4^+	30	Tris-HCl	8	5
16	1.5.1.-	Amine Dehydrogenase	AdRedAm (N-His) / C5GTJ9	*Blastomyces dermatitidis*	0.0121	0.0048	0.0121	1.00	0.40	1.00	**Hexanal** + Cyclo, 3-Buten-, **Methyl**-, Isopropyl **amine**	carbonyl compounds + prim. amine + NADPH -->reductive coupling + NADP+	42	Tris-HCl	7.5	1
17	1.5.1.48	Amine Dehydrogenase	IRED-(S)_Pe / WP_010497949.1	*Paenibacillus elgii*	0.3965	0.0040	0.3730	1.00	0.01	0.94	3,4-Dihydroisochinolin	3,4-Dihydroisochinolin + NADPH > 1,2,3,4-tatrahydroisoquinoline	30	Tris-HCl	7.5	1
18	1.6.99.1	Enoate Reductase	*Ts*ER (CrS) / B0JDW3	*Thermus scotoductus*	0.42	4.74	2.31	1.00	11.24	5.48	Cyclohexenone	Cyclohexenone + NAD(P)H <> Cyclohexanone + $NAD(P)^+$	30	Tris-HCl	8	5
19	1.14.13.22	Baeyer Villiger Monooxygenase	CHMO (BVMO) / P 12015	*Acinetobacter sp.*	4.15	0.17	3.94	1.00	-0.04	0.95	Cyclohexanone	Cyclohexanone + NADPH + H^+ + O_2 > caprolactone + $NADP^+$ + H_2O	30	Tris-HCl	8	2
20	1.14.13.84	Baeyer Villiger	HAPMO (BVMO) /	*Pseudomonas putida JD1*	0.79	0.09	0.87	1.00	0.12	1.10	(4-hydroxyphenyl)ethan-1-	(4-hydroxyphenyl)ethan-1-one + NADPH + H^+ + O_2 > 4-hydroxyphenyl	30	Tris-HCl	8	2

		Monooxygenase	Q88J44								one	acetate + $NADP^+$ + H_2O				
21	1.2.1.2.	Formate Dehydrogenase	Formate DH /O13437	*Candida boidinii*	9.42	2.10	0.53	1.00	0.22	0.06	Formate	Formate + NAD_+ <> CO_2 + NADH	30	KPi	7.5	5
23	1.1.1.1	Alcohol Dehydrogenase	ADH	Equine	0.0123	0.0203	0.0079	1.00	1.64	0.64	Butanal	Butanol + $NADP^+$ <> Butanal + NADPH	30	Tris-HCl	7.5	5

5. Supplementary References

(1) Zachos, I.; Genth, R.; Sutiono, S.; Marczynski, M.; Lieleg, O.; Sieber, V. Hot Flows: Evolving an Archaeal Glucose Dehydrogenase for Ultrastable Carba-NADP+ Using Microfluidics at Elevated Temperatures. *ACS Catalysis* **2022**, *12* (3), 1841-1846. DOI: 10.1021/acscatal.1c04320.

(2) Szczepankiewicz, B. G.; Dai, H.; Koppetsch, K. J.; Qian, D.; Jiang, F.; Mao, C.; Perni, R. B. Synthesis of Carba-NAD and the Structures of Its Ternary Complexes with SIRT3 and SIRT5. *The Journal of Organic Chemistry* **2012**, *77* (17), 7319-7329. DOI: 10.1021/jo301067e.

(3) Slama, J. T.; Simmons, A. M. Carbanicotinamide adenine dinucleotide: synthesis and enzymological properties of a carbocyclic analogue of oxidized nicotinamide adenine dinucleotide. *Biochemistry* **1988**, *27* (1), 183-193. DOI: 10.1021/bi00401a028 From NLM.

(4) Hutchinson, E. J.; Taylor, B. F.; Blackburn, G. M. Synthesis of carbocyclic NAD+ containing a methylenebisphosphonate linkage for the investigation of ADP-ribosyl cyclase. *Chemical Communications* **1996**, (24), 2765-2766, 10.1039/CC9960002765. DOI: 10.1039/CC9960002765.

(5) Kurnasov Oleg, V.; Polanuyer Boris, M.; Ananta, S.; Sloutsky, R.; Tam, A.; Gerdes Svetlana, Y.; Osterman Andrei, L. Ribosylnicotinamide Kinase Domain of NadR Protein: Identification and Implications in NAD Biosynthesis. *Journal of Bacteriology* **2002**, *184* (24), 6906-6917. DOI: 10.1128/jb.184.24.6906-6917.2002 (acccessed 2024/10/24).

(6) Balducci, E.; Emanuelli, M.; Raffaelli, N.; Ruggieri, S.; Amici, A.; Magni, G.; Orsomando, G.; Polzonetti, V.; Natalini, P. Assay Methods for Nicotinamide Mononucleotide Adenylyltransferase of Wide Applicability. *Analytical Biochemistry* **1995**, *228* (1), 64-68. DOI: https://doi.org/10.1006/abio.1995.1315.

(7) Ohashi, K.; Kawai, S.; Murata, K. Identification and characterization of a human mitochondrial NAD kinase. *Nature Communications* **2012**, *3* (1), 1248. DOI: 10.1038/ncomms2262.
